# Combining a nanoparticle-mediated immunoradiotherapy with dual blockade of LAG3 and TIGIT improves the treatment efficacy in anti-PD1 resistant lung cancer

**DOI:** 10.1186/s12951-022-01621-4

**Published:** 2022-09-19

**Authors:** Yun Hu, Sébastien Paris, Genevieve Bertolet, Hampartsoum B. Barsoumian, Kewen He, Duygu Sezen, Dawei Chen, Mark Wasley, Jordan DA SILVA, Joylise A. Mitchell, Tiffany A. Voss, Fatemeh Masrorpour, Claudia Kettlun Leyton, Liangpeng Yang, Carola Leuschner, Nahum Puebla-Osorio, Saumil Gandhi, Quynh-Nhu Nguyen, Maria Angelica Cortez, James W. Welsh

**Affiliations:** 1 Department of Radiation Oncology, Unit 97, The University of Texas MD Anderson Cancer, 1515 Holcombe Blvd, Houston, TX 77030 USA; 2 Department of Translational Science, Nanobiotix, Paris, France; 3 Department of Radiation Oncology, Shandong Cancer Hospital and Institute, Shandong First Medical University, Shandong Academy of Medical Sciences, Jinan, China; 4 Department of Radiation Oncology, Koc University School of Medicine, Istanbul, Turkey

**Keywords:** Nanoparticle, NBTXR3, Immunoradiotherapy, Radiotherapy, Checkpoint blockade, Anti-PD1 resistance

## Abstract

**Background:**

While improvements in immunoradiotherapy have significantly improved outcomes for cancer patients, this treatment approach has nevertheless proven ineffective at controlling the majority of malignancies. One of the mechanisms of resistance to immunoradiotherapy is that immune cells may be suppressed via the myriad of different immune checkpoint receptors. Therefore, simultaneous blockade of multiple immune checkpoint receptors may enhance the treatment efficacy of immunoradiotherapy.

**Methods:**

We combined NBTXR3-enhanced localized radiation with the simultaneous blockade of three different checkpoint receptors: PD1, LAG3, and TIGIT, and tested the treatment efficacy in an anti-PD1-resistant lung cancer model in mice. 129 Sv/Ev mice were inoculated with fifty thousand αPD1-resistant 344SQR cells in the right leg on day 0 to establish primary tumors and with the same number of cells in the left leg on day 4 to establish the secondary tumors. NBTXR3 was intratumorally injected into the primary tumors on day 7, which were irradiated with 12 Gy on days 8, 9, and 10. Anti-PD1 (200 µg), αLAG3 (200 µg), and αTIGIT (200 µg) were given to mice by intraperitoneal injections on days 5, 8, 11, 14, 21, 28, 35, and 42.

**Results:**

This nanoparticle-mediated combination therapy is effective at controlling the growth of irradiated and distant unirradiated tumors, enhancing animal survival, and is the only one that led to the destruction of both tumors in approximately 30% of the treated mice. Corresponding with this improved response is robust activation of the immune response, as manifested by increased numbers of immune cells along with a transcriptional signature of both innate and adaptive immunity within the tumor. Furthermore, mice treated with this combinatorial therapy display immunological memory response when rechallenged by the same cancer cells, preventing tumor engraftment.

**Conclusion:**

Our results strongly attest to the efficacy and validity of combining nanoparticle-enhanced radiotherapy and simultaneous blockade of multiple immune checkpoint receptors and provide a pre-clinical rationale for investigating its translation into human patients.

**Graphical Abstract:**

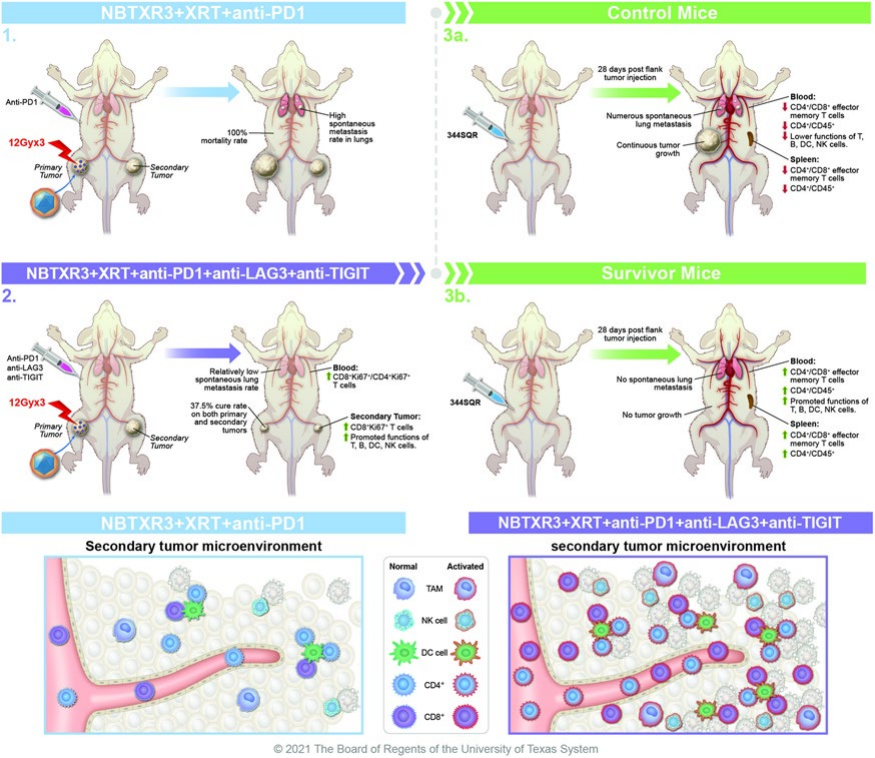

**Supplementary Information:**

The online version contains supplementary material available at 10.1186/s12951-022-01621-4.

## Introduction

In the past decade, great strides have been made in cancer therapy with the ascendancy of immune checkpoint inhibitors (CPIs). These drugs work by using blocking antibodies to disrupt the inhibitory signaling of immune checkpoint proteins that reside on the surface of lymphocytes such as T cells and natural killer (NK) cells. These immune checkpoint receptors (ICRs) include a wide array of different proteins, including CTLA4, PD1, LAG3, TIGIT, and still others [1–5]. The first CPI to be approved for clinical use was ipilimumab—which targets CTLA4—in 2011 [6]. Three years later, in 2014, two PD1 inhibitors, nivolumab and pembrolizumab were approved, followed swiftly by approval of inhibitors against the ligand for PD1, PD-L1, in 2015 (avelumab), 2016 (atezolizumab), and 2017 (durvalumab) [6]. Together, these drugs have revolutionized the treatment of certain cancers, with particular success in melanoma and hematological malignancies. As a result of these successes, CPI has become standard-of-care therapy for a host of cancer types.

Despite these advances, however, the grim reality is that the majority of patients do not even respond to these treatments, let alone experience durable remission [7]. One of the major culprits underlying this intractability is that cancers upregulate a slew of ligands for the myriad of different ICRs that immune cells express [8]. Thus, if one is inhibited, others become upregulated in its place to compensate. Simultaneously targeting multiple ICRs may be a viable strategy to circumvent this limitation [9, 10]. Accordingly, the field has sought other CPIs, with Phase I/II clinical trials ongoing for novel inhibitors against many of the previously mentioned ICRs.

However, in the perpetual war on cancer, CPIs are not the only weapons being developed. Significant and exciting improvements have been made in the oldest anti-cancer treatment besides surgical resection, radiation therapy (RT). The combination of radiotherapy and immune checkpoint inhibition (immunoradiotherapy, or IRT) has been widely considered promising in various cancers [11, 12]. IRT can effectively educate the immune system to target and destroy cancer cells locally and systemically. However, its efficacy remains limited in many types of cancers, particularly in those resistant to the treatment of CPIs [13–15]. For instance, the majority of lung cancer patients do not respond to anti-PD1 (αPD1) treatment, and these patients are most likely insensitive to the combination of radiation and αPD1 treatment [16].

One notable recent advance in IRT has been the development of radio-enhancing metal nanoparticles. These nanoscale, electron-dense particles can be injected into the tumor and then stimulated with ionizing radiation, causing the amplified release of their energy payloads and delivering a highly focused radiation dose directly to the tumor. One such nanoparticle, NBTXR3, developed by Nanobiotix [17], has shown promising results in the clinic and, in light thereof, has been approved for the treatment of localized soft-tissue sarcomas in Europe [18, 19].

To improve the therapeutic outcome of IRT in αPD1-resistant tumors, we previously incorporated NBTXR3 in our IRT regimen, consisting of localized radiation and systemic αPD1 injection for treating αPD1-resistant lung cancer (344SQR) in mice. We found that NBTXR3 significantly enhanced the control of both the irradiated primary tumor and unirradiated distant tumor, thereby extending the survival of the mice [20, 21]. Further investigation revealed that NBTXR3-mediated IRT considerably upregulated the activities of major antitumor immune pathways and facilitated the infiltration of CD8^+^ T cells into the unirradiated tumors. This satellite action is known as the abscopal effect [22], and it has also been observed following NBTXR3 treatment by other groups [23]. Despite these promising results, this combination therapy was not potent enough to completely eliminate the tumors. We also found that triple therapy of NBTXR3, XRT, and αPD1 significantly upregulated the expression of Lymphocyte Activation Gene 3 (LAG3) and Tyrosine-based inhibitory motif domain (TIGIT) in both irradiated and unirradiated tumors (Additional file 1: Fig. S1). LAG3 and TIGIT can deliver inhibitory signals that regulate immune cell homeostasis, T cell activation, and proliferation [24]. Substantial evidence supports the notion that both LAG3 and TIGIT contribute to the exhaustion of CD4^+^, CD8^+^ T cells, and NK cells and limit the adaptive and innate immune response against tumors [25]. In addition, ample clinical and preclinical results demonstrate that blockade of TIGIT and LAG3 with antibodies reinvigorates exhausted CD8^+^ T cells and significantly improves antitumor immune responses [26–28].

Therefore, to further improve the treatment efficacy of NBTXR3-mediated IRT in αPD1-resistant lung cancer, αPD1, αLAG3, and αTIGIT monoclonal antibodies were co-injected into mice, and the primary tumors were given NBTXR3-enhanced RT. Adding αLAG3 or αTIGIT alone to the combination of NBTXR3 + XRT + αPD1 significantly improved the control of both the primary and secondary tumors. More profoundly, the co-blockade of LAG3 in tandem with TIGIT affected additional tumor control, translating to a survival rate of 37.5% in mice. This study demonstrates a highly effective combination therapy involving nanoparticle-enhanced radiotherapy, traditional αPD1, and the novel immune CPIs αTIGIT and αLAG3. This novel combination—and the results garnered therewith—provide a promising IRT strategy for treating αPD1-resistant tumors in the clinic.

## Materials and methods

### Materials

Nanobiotix provided radiation-enhancing nanoparticles (NBTXR3). Bristol-Myers Squibb provided αPD1, αLAG3, and αTIGIT. Antibodies for flow cytometry, including αCD45-APC-Cy7 (cat. #103116), αCD3-PE-Cy7 (cat. #100220), αCD4-alexa 700 (cat. #100430), αCD8-PercpCy5.5 (cat. #100734), αCD49b-PE (cat. #108908), αKi67-alexa 647 (cat. #652408), αCD45-Pacific Blue (cat. #103126), αCD3-BV510 (cat. #100234), αCD4-APC-Fire 750 (cat. #100568), αCD62L-PE-Cy7 (cat. #104418), and αCD44-APC (cat. #103012) were purchased from Biolegend. Bouin’s fixative solution (cat. #16045-1) was purchased from Polysciences Inc. Trizol (Cat. #15596018) was purchased from Thermo Fisher Scientific. Liberase (cat. #05401127001) and DNAse (cat. #4716728001) were purchased from Roche and Sigma-Aldrich, respectively. The depletion antibodies, including, αCD4 (cat. #BE0003-1), αCD8 (cat. #BE0004-1) were purchased from BioXcell, and αAsialo GM1 (cat. #986-10001) was purchased from FUJIFILM Wako Chemicals USA, Corp.

### Cell lines and culture conditions

344SQR, an αPD1-resistant lung cancer cell line [29], was used for all in vivo experiments. The 344SQR cells were cultured in RPMI-1640 medium supplemented with 10% fetal bovine serum and penicillin/streptomycin and incubated at 37 °C in a 5% CO_2_ atmosphere.

### Tumor establishment and combination therapy

**Fig. 1:**
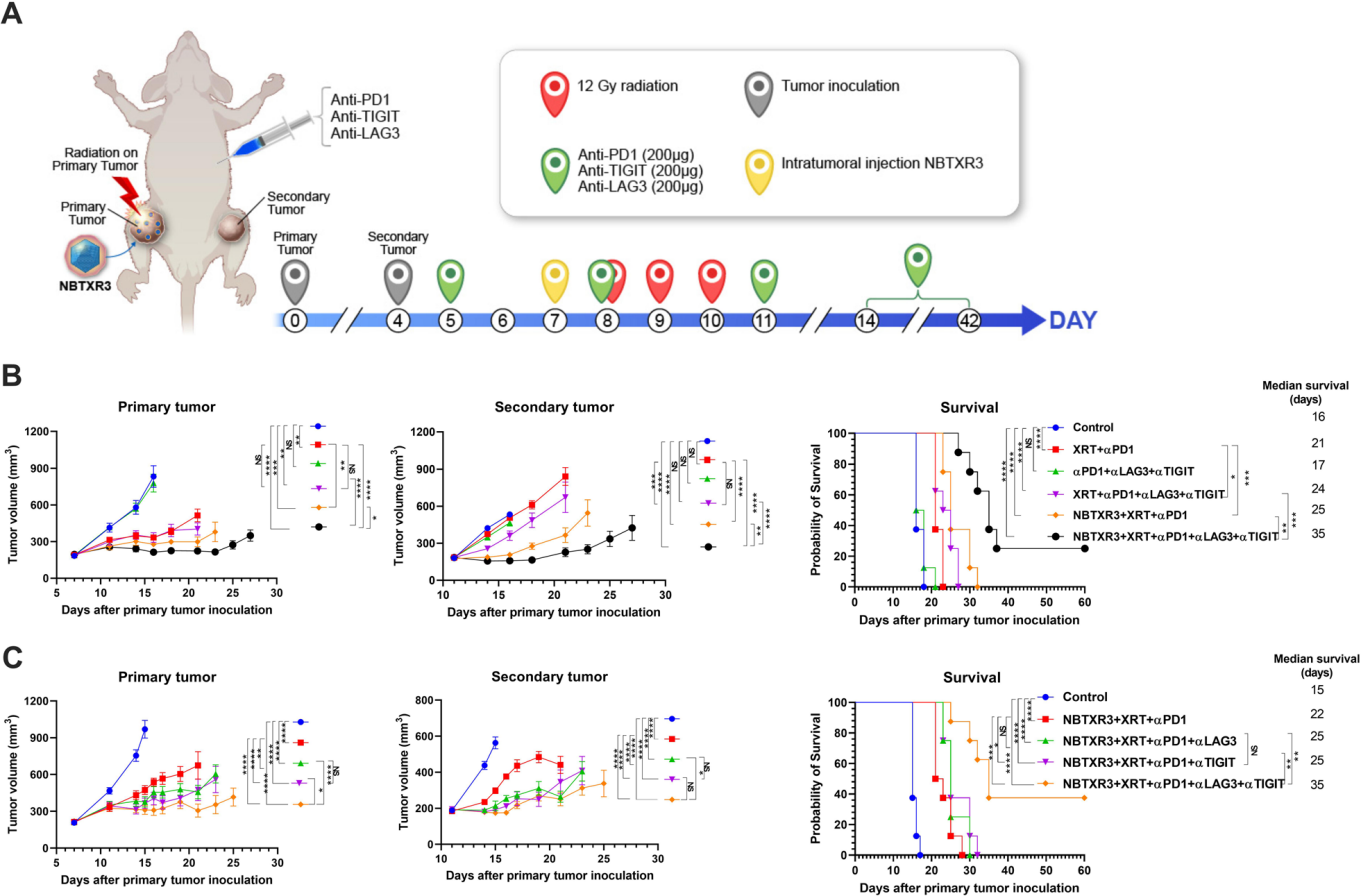
Dual blockade of LAG3 and TIGIT improves treatment outcome of NBTXR3-mediated immunoradiotherapy. **A** Treatment scheme. 129/SvEv syngeneic female mice (8–12 weeks old) were inoculated with 344SQR cells on the right leg and left leg to establish primary and secondary tumors, respectively. The primary tumors were intratumorally injected with NBTXR3 on day 7, followed by three fractions of 12 Gy radiation on days 8, 9, and 10. αPD1 (200 µg), αLAG3 (200 µg), and αTIGIT (200 µg) were administered at the indicated time points *via* intraperitoneal injection. The tumor size was monitored, and the mice were euthanized when the tumor dimension reached 14 mm. **B** Tumor volume of the mice (n = 8) receiving the indicated treatment and survival curve. **C** Tumor volume and survival rate of the mice (n = 8) for comparing the efficacy of αLAG3, αTIGIT, and their combination when added to NBTXR3 + XRT + αPD1. Tumor volumes were compared by two-way analysis of variance and were expressed as mean tumor volume ± standard error of the mean ± SEM. Mouse survival rates were analyzed with the Kaplan–Meier method. *P* < 0.05 was considered statistically significant. *P < 0.05, **P < 0.01, ***P < 0.001, ****P < 0.0001, NS, not significant

Eight- to 12-week-old 129/SvEv syngeneic female mice from Taconic Biosciences were used in this study. As shown in Fig. 1A, the 344SQR cells (5 × 10^4^ in 100 µL phosphate-buffered saline [PBS]) were subcutaneously injected into the right legs of the mice on day 0 to establish the “primary” tumor (to be irradiated) and into the left legs on day 4 to establish the “secondary” tumor. Tumors were measured starting from day seven following injection of the first leg, and the tumor volumes were calculated as V = 0.5 × width^2^ × length. All the mice were given intraperitoneal injections of various combinations of CPI antibodies: αPD1 (200 µg), αTIGIT (200 µg), and αLAG3 (200 µg) on days 5, 8, 11, 14, 21, 28, 35, and 42. The primary tumors were intratumorally injected with 25% tumor volume of NBTXR3 in 5% glucose on day 7, followed by three fractions of 12 Gy radiation with a PXi X-Rad SmART irradiator on days 8, 9, and 10. Mice were euthanized when the primary or the secondary tumors reached 14 mm in any dimension. All animal procedures followed the guidelines of the Institutional Animal Care and Use Committee at The University of Texas MD Anderson Cancer Center.

### Depletion of CD4^+^ T cells, CD8^+^ T cells, and NK cells

As described above, the primary and the secondary tumors were inoculated with 344SQR cells in mice (n = 5). The mice were treated with the combination therapy of NBTXR3 + XRT + αPD1 + αLAG3 + αTIGIT as described above. αCD4 (500 µg), αCD8 (500 µg), and αAsialo GM1 (30 µL) antibodies were given *via* intraperitoneal injection on days 5, 7, 9, 12, and 17 to deplete CD4^+^ T cells, CD8^+^ T cells, and NK cells, respectively. Mice were euthanized when the primary or the secondary tumors reached 14 mm in any dimension.

### Tumor re-challenge

The survivor mice from NBTXR3 + XRT + αPD1 + αLAG3 + αTIGIT (NBTXR3 + XRT + PLT) group were given subcutaneous injections of 5 × 10^4^ 344SQR cells in 100 µL PBS in the right flank at least 60 days post radiotherapy. Five untreated mice were injected with the same amount of 344SQR cells and served as the control. No further treatment was given. All mice were euthanized 28 days after tumor inoculation, and lungs, spleen, and blood samples were harvested to count the numbers of lung metastases and to obtain profiles of CD4^+^ and CD8^+^ memory T cells.

### Lung metastasis analysis

Lungs were harvested on day 21, or, in the tumor re-challenge experiment, from both the Control group and the NBTXR3 + XRT + PLT group 28 days after the re-challenge. The harvested lungs were stored in Bouin’s fixative solution for three days, after which lung metastatic nodules were counted.

### Tumor processing

Both the primary and the secondary tumors were harvested on day 21. The minced tumor tissues were digested with 250 µg/mL of liberase and 20 µg/mL DNAse at 37 °C for 30 min. The digestion process was stopped with 1 mL fetal bovine serum, and the samples were filtered. The cells were either stained for flow cytometry analysis or frozen in TRIzol for RNA extraction.

### Flow cytometry analysis

The cells processed from the tumor tissue were stained with αCD45-APC-Cy7, αCD3-PE-Cy7, αCD4-alexa 700, αCD8-PercpCy5.5, and αKi67-alexa 647. Splenocytes and blood samples from the tumor re-challenge experiment were stained with αCD45-Pacific Blue, αCD4-APC-Fire 750, αCD8-PercpCy5.5, αCD62L-PE-Cy7, and αCD44-APC. Samples were run with a Gallios Flow Cytometer (Beckman Coulter) and analyzed with Kaluza software version 2.1.

### RNA extraction and nanostring analysis of immune-related genes

Total RNA was extracted from both the primary and the secondary tumors or blood with the chloroform/phenol method [30]. RNA quality and quantity were assessed with a Nanodrop spectrophotometer (Thermo Scientific, Waltham, MA). At least 50 ng of the extracted RNA was analyzed with a nCounter PanCancer Immune Profiling Panel and a nCounter MAX Analysis System (both from NanoString Technologies, Seattle, WA, USA) according to the manufacturer’s instructions; data were processed with the PanCancer Immune Profiling Advanced Analysis Module (also from NanoString Technologies).

### Statistical analyses

All statistical analyses were performed with Prism 8.0 (GraphPad Software). Tumor growth curves were compared by two-way analysis of variance and were expressed as mean tumor volume ± standard error of the mean (SEM). Mouse survival rates were analyzed with the Kaplan–Meier method, and estimates were compared with log-rank tests. All other data were analyzed with two-tailed *t* tests and expressed as mean value ± SEM. P values of < 0.05 were considered to indicate statistically significant differences.

## Results

### Dual blockade of LAG3 and TIGIT improves treatment outcome of NBTXR3-mediated immunoradiotherapy

To address the upregulation of LAG3 and TIGIT induced by the treatment of NBTXR3 + XRT + αPD1, we established a two-tumor model with 344SQR αPD1-resistant lung cancer in mice, which were subsequently treated with various combinations of radiation (XRT), XRT enhanced with NBTXR3, αPD1, αLAG3, and αTIGIT (Fig. 1A). Consistent with our previously published results [20], irradiation of tumors injected with NBTXR3 and treated with αPD1 produced superior control of tumor growth and longer animal survival time than XRT + αPD1 without nanoparticle injection (Fig. 1B). The combination of triple checkpoint blockade (αPD1 + αLAG3 + αTIGIT, hereafter abbreviated PLT) in the absence of any radiotherapy (XRT alone or NBTXR3 + XRT) did not achieve significant control of either the primary or the secondary tumors. In addition, the co-blockade of LAG3 and TIGIT did not enhance treatment outcomes of XRT + αPD1 without NBTXR3. However, adding NBTXR3 + XRT + PLT led to significantly slower growth of both the primary and the secondary tumors as well as extended survival. The median survival times of each group, in days, were as follows: control (16), XRT + αPD1 (21), PLT (17), XRT + PLT (24), NBTXR3 + XRT + αPD1 (25), and NBTXR3 + XRT + PLT (35) (Fig. 1B, C). NBTXR3 + XRT + PLT markedly slowed the tumor growth in most of the treated mice, and in 25% (2 out of 8) of the mice that received NBTXR3 + XRT + PLT, the tumors were completely eradicated (Additional file 1: Fig. S2). In contrast, no mice from any of the other treatment groups survived the entire assay. Although NBTXR3 + XRT + αPD1 was effective in delaying the growth of both the primary and secondary tumors in most of the mice, it was ultimately unable to stop tumor growth in any of them.

Having established the superiority of NBTXR3 + XRT + PLT, we next sought to evaluate the benefit of adding either αLAG3 or αTIGIT individually to NBTXR3 + XRT + αPD1. Either αTIGIT or αLAG3 was able to significantly improve control of both the primary and the secondary tumors, and no significantly different treatment efficacy was observed between NBTXR3 + XRT + αPD1 + αLAG3 and NBTXR3 + XRT + αPD1 + αTIGIT in terms of tumor growth or survival (Fig. 1C). However, neither of these two combination therapies achieved remission of the tumors. In contrast, in this particular survival assay, treatment with NBTXR3 + XRT + PLT resulted in 3 of the 8 mice (37.5%) being entirely cured from their tumors (Fig. 1C).

Previously, we observed that improved control of primary and secondary tumors was accompanied by fewer lung metastases [20]. To confirm this result in our present study, we counted the number of metastatic lesions in the lungs of our mice on day 21. In keeping with our prior observations, lung metastasis corresponded sharply with control of the primary and secondary tumors (Additional file 1: Fig. S3). Every treatment group paired NBTXR3 with any combination of CPIs significantly reduced the number of spontaneous lung metastases compared to the control. The addition of either αLAG3 or αTIGIT to NBTXR3 + XRT + αPD1 resulted in significantly fewer lung metastases, and the addition of both in concert achieved the lowest numbers of metastases of any treatment group.

Lastly, we monitored the body weight of the mice implanted with 344SQR tumors followed by treatment with NBTXR3 + XRT + PLT and those untreated naïve mice, no significant difference in body weight was observed between the two groups (Additional file 1: Fig. S4).

### The treatment efficacy of NBTXR3 + XRT + CPIs is heavily dependent on immune cells

**Fig. 2:**
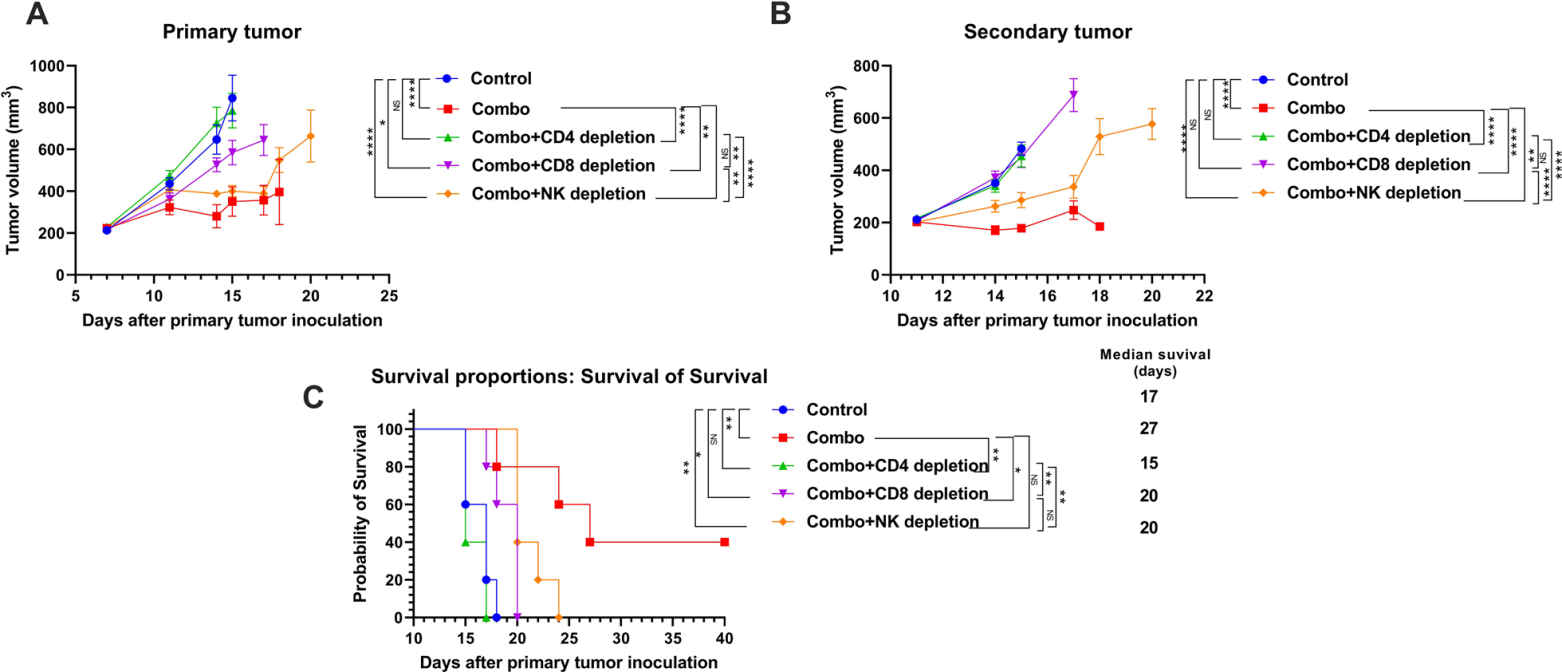
The treatment efficacy of NBTXR3 + XRT + PLT (Combo) heavily depends on immune cells. Mice (n = 5) were treated by the combination therapy of NBTXR3 + XRT + PLT, as described in Fig. 1. In addition, αCD4 (500 µg), αCD8 (500 µg), and αAsialo GM1 (30 µL) antibodies were given *via* intraperitoneal injection on days 5, 7, 9, and 12, and 17 to deplete CD4^+^ T cells, CD8^+^ T cells, and NK cells, respectively. Mice were euthanized when the primary or the secondary tumors reached 14 mm in any dimension. **A** Tumor volume of the primary tumor. **B** Tumor volume of the secondary tumor. **C** Survival rates of the mice. Tumor volumes were compared by two-way analysis of variance and were expressed as mean tumor volume ± standard error of the mean ± SEM. Mouse survival rates were analyzed with the Kaplan–Meier method. *P* < 0.05 was considered statistically significant. *P < 0.05, **P < 0.01, ***P < 0.001, ****P < 0.0001, NS, not significant

The abscopal effect is thought to be mediated by the immune response [22]. To elucidate if the treatment benefits we observed in our NBTXR3 + XRT + PLT treatment group were indeed immune-mediated and, if so, what populations of immune cells were involved in the antitumor activity, we depleted CD4^+^ T cells, CD8^+^ T cells, and NK cells with antibodies from mice in this treatment group. The depletion of CD4^+^ T cells, CD8^+^ T cells, and NK cells all detrimentally impacted the treatment efficacy of the NBTXR3 + XRT + PLT therapy, but at different levels (Fig. 2). CD4^+^ T cell depletion completely ablated the tumor control efficacy of the NBTXR3 + XRT + PLT therapy, resulting in tumor growth and survival curves virtually indistinguishable from those of untreated controls (Fig. 2A–C). Depletion of CD8^+^ T cells impaired but did not entirely ablate control of primary tumor growth and overall survival in the NBTXR3 + XRT + PLT group; like with CD4^+^ depletion, however, secondary tumor growth was completely unrestrained. NK cell depletion had the least impact, with primary tumor growth curves only slightly inferior to treated mice who were not immunodepleted; the difference in secondary tumor volume was more substantial, but still the least affected of the immuno-depleted treatment groups. The survival time of NK-depleted mice was slightly improved over those of CD8-depleted. In any case, all immunodepletion interventions impaired the treatment efficacy of NBTXR3 + XRT + PLT. Two out of the 5 mice from the non-immunodepleted group survived the entire experiment; none of the others did. Our results demonstrate that CD4^+^ T cells, CD8^+^ T cells, and NK cells are all essential to the efficacy of the NBTXR3 + XRT + PLT treatment, with CD4^+^ T cells being the most indispensable and NK cells the least.

### Dual blockade of LAG3 and TIGIT increases proliferation of CD4^+^ and CD8^+^ T cells

By now, we had established two points: that mice treated with NBTXR3 + XRT + PLT could control tumor growth and survive in a way that XRT + PLT treated mice were unable to match, and that this ability was dependent upon T cells. Given these two points, we decided to examine what effect this treatment might have on these immune cell populations. TIGIT and LAG3 are well-known to induce T cell exhaustion. Thus, we reasoned that these two markers’ blockade might alleviate T cell exhaustion.

**Fig. 3:**
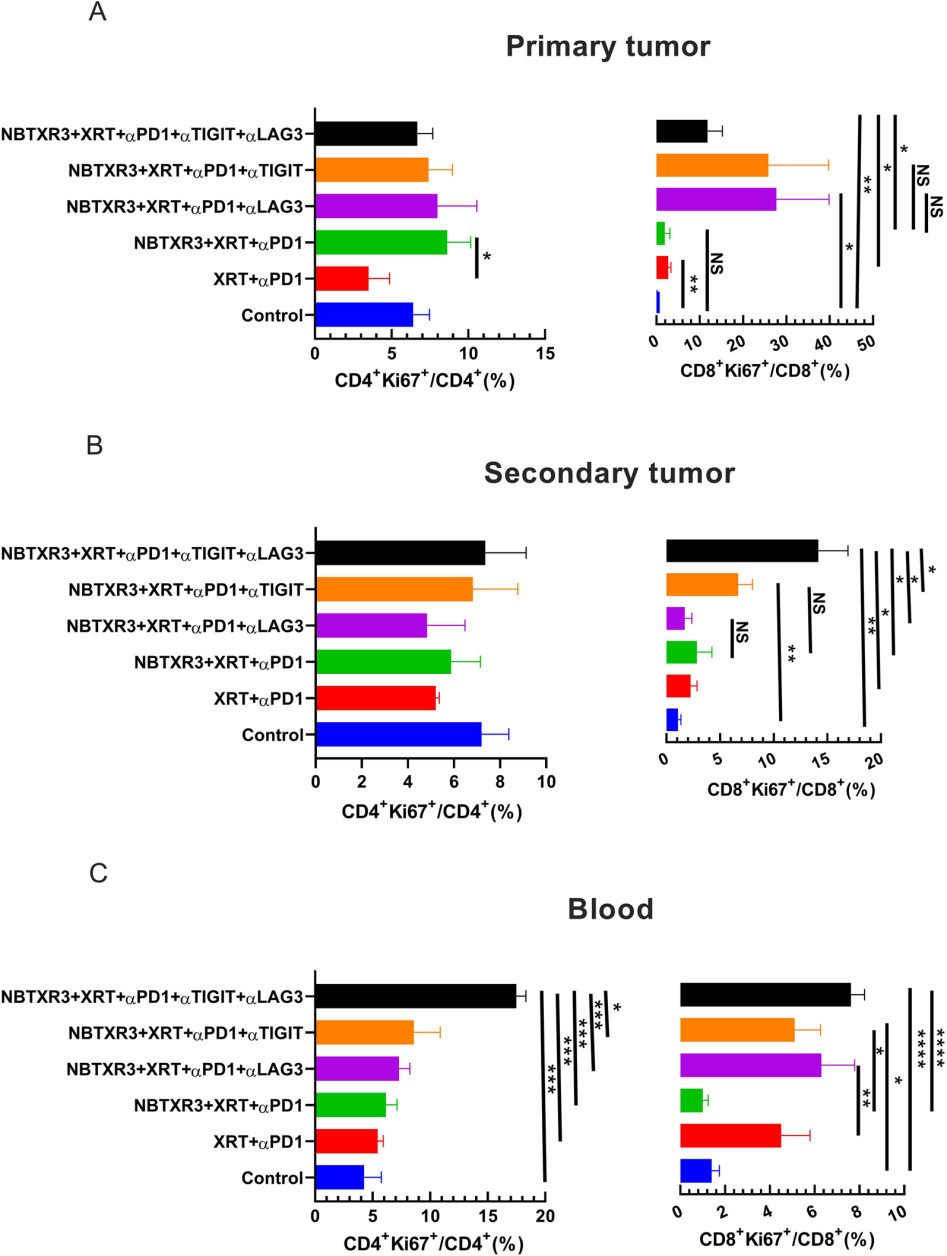
Dual blockade of TIGIT and LAG3 promotes proliferation of CD4^+^ and CD8^+^ T cells. **A** Percentages of Ki67^+^CD4^+^ and Ki67^+^CD8^+^ T cells in the primary tumors. **B** Percentages of Ki67^+^CD4^+^ and Ki67^+^CD8^+^ T cells in the secondary tumors. **C** Percentages of Ki67^+^CD4^+^ and Ki67^+^CD8^+^ T cells in the blood. The mice (n = 5) were treated with various combination therapies, including XRT + αPD1, NBTXR3 + XRT + αPD1, NBTXR3 + XRT + αPD1 + αLAG3, NBTXR3 + XRT + αPD1 + αTIGIT, and NBTXR3 + XRT + PLT as indicated in Fig. 1 A and were sacrificed on day 21. The mice which were inoculated with tumors only served as control. Immune cells from primary tumors, secondary tumors, and blood were processed and stained with αCD45-APC-Cy7, αCD3-PE-Cy7, αCD4-alexa 700, αCD8-PercpCy5.5, and αKi67-alexa 647. The cells were run with a Gallios Flow Cytometer (Beckman Coulter) and analyzed with Kaluza software Version 2.1. The data were expressed as mean ± SEM and were analyzed with a two-tailed t test. *P* < 0.05 was considered statistically significant. *P < 0.05, **P < 0.01, ***P < 0.001, ****P < 0.0001

To evaluate this hypothesis, we analyzed the proliferation of both CD4^+^ and CD8^+^ T cells, as assessed by Ki67 expression, in mice treated with XRT + αPD1, NBTXR3 + XRT + αPD1, NBTXR3 + XRT + αPD1 + αLAG3, NBTXR3 + XRT + αPD1 + αTIGIT, and NBTXR3 + XRT + PLT. No significant differences were detected in the percentage of CD4^+^Ki67^+^/CD4^+^ T cells in the primary or the secondary tumors between the experimental groups (Fig. 3A, B and Additional file 1: Fig. S5A, B, and D). In contrast, proliferating CD8^+^ T cells increased in the primary tumors of mice treated with αTIGIT and/or αLAG3 (Fig. 3 A and Additional file 1: Fig. S5A), a significantly higher percentage of CD8^+^Ki67^+^ T cells was observed in the secondary tumors in mice treated with either αTIGIT (but not αLAG3), and a much greater percentage was observed in the secondary tumors of mice treated with NBTXR3 + XRT + PLT (Fig. 3B and Additional file 1: Fig. S5B). In the blood, flow cytometry analysis show that NBTXR3 + XRT + PLT produced a significantly higher percentage of Ki67^+^CD4^+^ T cells than all other combination therapies and a significantly higher percentage of Ki67^+^CD8^+^ T cells than the NBTXR3 + XRT + αPD1 group, but not the NBTXR3 + XRT + αPD1 + αLAG3 or NBTXR3 + XRT + αPD1 + αTIGIT group (Fig. 3C and Additional file 1: Fig. S5C). These results show that dual blockade of LAG3 and TIGIT, in concert with NBTXR3-amplified radiation therapy and PD1 blockade, promotes the proliferation of intratumoral proliferation of CD8^+^ T cells and the systemic proliferation of both CD4^+^ and CD8^+^ T cells. In addition, nanostring analysis of dual blockade of LAG3 and TIGIT did not significantly change the number of CD8^+^ T, NK, B, and Treg cells in the primary or secondary tumors. However, the mice treated with NBTXR3 + XRT + PLT exhibited more CD45^+^ immune cells in the secondary tumors (Additional file 1: Fig. S7).

### NBTXR3, in tandem with triple CPIs, stimulates the activation of immunological genetic programs

**Fig. 4:**
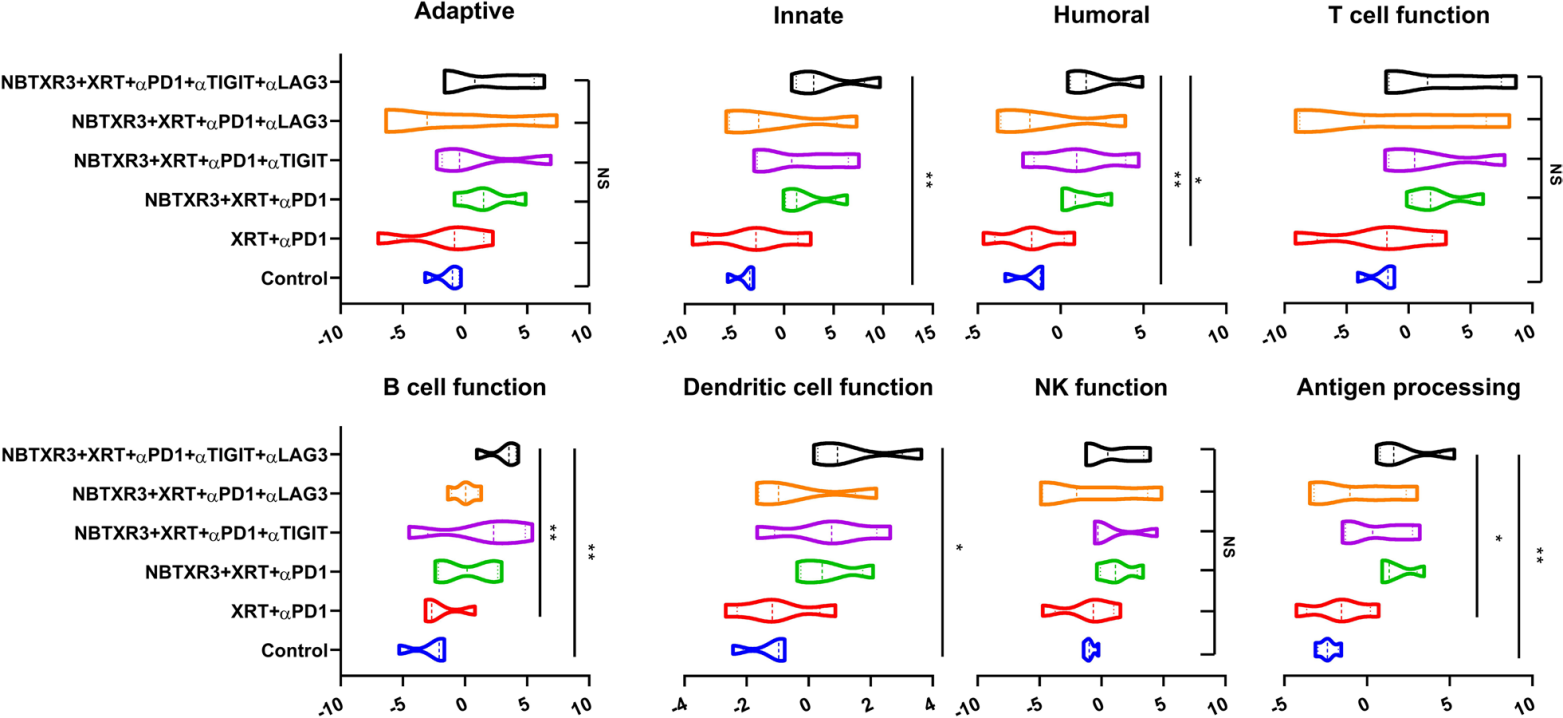
Activity score of immune pathways in the irradiated tumors. Mice (n = 4) were treated with various combination therapies as described in Fig. 1 and were euthanized 11 d post last fraction of radiation. The RNA from the irradiated tumors was extracted, and the immune-related genes were measured with a nCounter PanCancer Immune Profiling Panel and a nCounter MAX Analysis System. The data were analyzed with the PanCancer Immune Profiling Advanced Analysis Module. *P* < 0.05 was considered statistically significant. *P < 0.05, **P < 0.01, ***P < 0.001, NS, not significant

Having delineated the efficacy of these combinatorial treatments in our mouse model, we next sought to parse the genetic responses to each treatment. To this end, we excised primary tumors from the mice and subjected them to complete cellular dissolution, followed by RNA extraction. We then analyzed the comparative abundance of 770 different immune-related genes using the Nanostring PanCancer Immune Profiling Advanced Analysis Module. We observed significant elevations in the expression levels of genes involved in innate immunity, humoral immunity, B cell function, DC function, and antigen processing in mice that were treated with NBTXR3 + XRT + PLT when compared to the control (Fig. 4). We also observed elevations in genes involved in adaptive immunity, T cell function, and NK cell function; however, these increases were not statistically significant. Similar elevations were observed for NBTXR3 + XRT + αPD1 and αLAG3 or NBTXR3 + XRT + αPD1 and αTIGIT, though these were not significantly higher than treatment with just NBTXR3 + XRT + αPD1. Remarkably, NBTXR3 + XRT + PLT also displayed increased activities in humoral, B cell function, and antigen processing pathways compared to XRT + αPD1(Fig. 4).

**Fig. 5:**
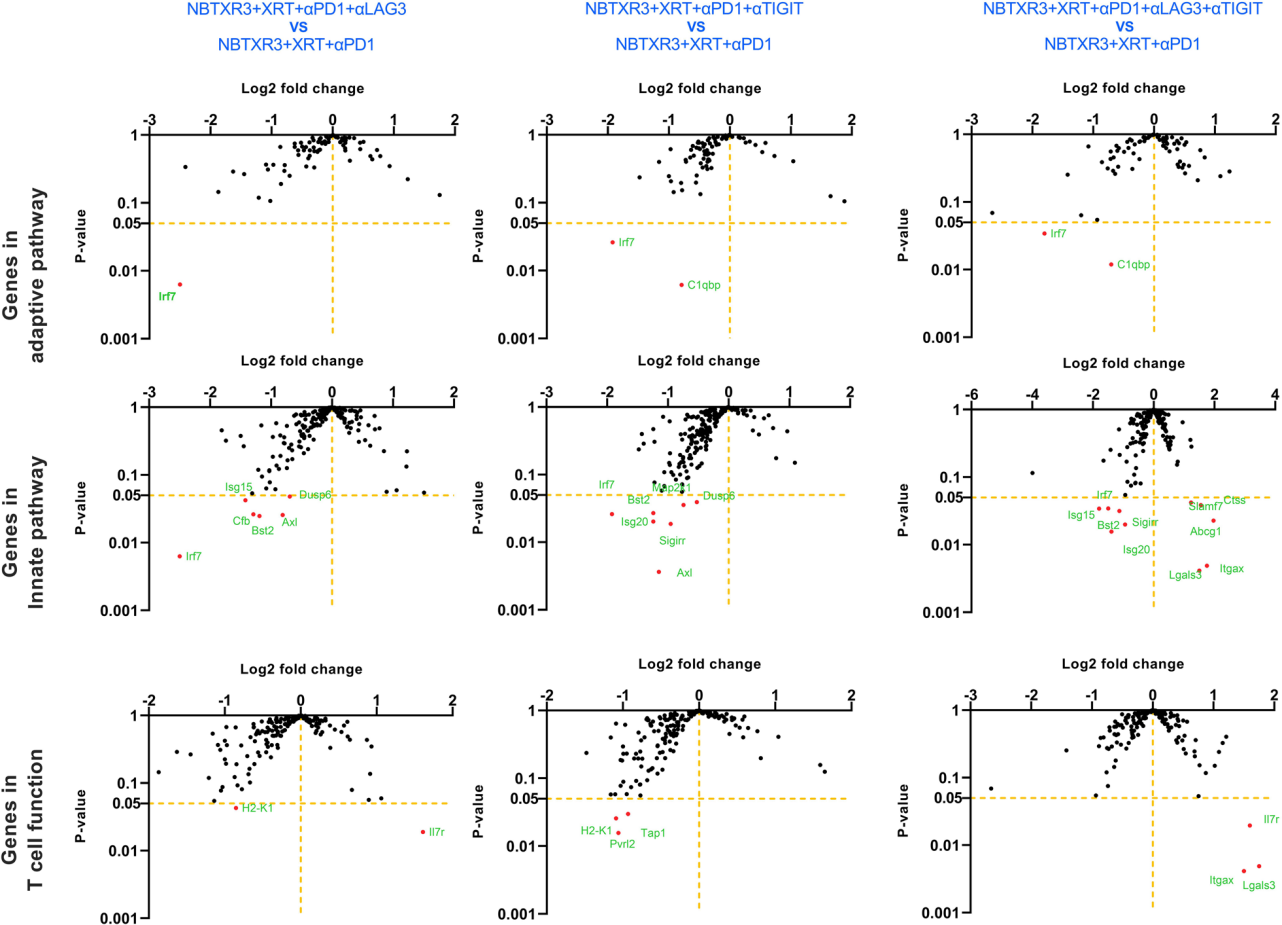
Log_2_ fold change in expression of genes involved in adaptive, innate immunity, and T cell function in the irradiated tumors. Mice (n = 4) were treated with various combination therapies as described in Fig. 1 and were euthanized 11 d post last fraction of radiation. The RNA from the irradiated tumors was extracted, and the immune-related genes were measured with a nCounter PanCancer Immune Profiling Panel and a nCounter MAX Analysis System. The data were analyzed with the PanCancer Immune Profiling Advanced Analysis Module

Given the unique efficacy we observed for NBTXR3 + XRT + PLT treatment, we specifically compared the immunogenetic profile of primary tumors treated with NBTXR3 + XRT + PLT, NBTXR3 + XRT + αPD1 and αLAG3, or NBTXR3 + XRT + αPD1 and αTIGIT as compared to those treated with NBTXR3 + XRT + αPD1 alone. In this manner, we hoped to see if each iterative addition of blockers induced any additional genetic activation. When we thus analyzed the RNA data, we observed no additional upregulation of adaptive immune-related genes with any combination of blockers above that achieved by NBTXR3 + XRT + αPD1 (Fig. 5). On the contrary, there was, in fact, a marked downregulation of two immune-related genes: *Irf7*, which is strongly involved in antiviral immunity [30], and *C1qbp*, a component of the complement protein C1q-binding receptor that is strongly associated with the promotion of chemotaxis and metastasis in several cancer types [31, 32].

When we examined innate immune genes, we saw significant downregulation of even more immune-related genes, all broadly associated with activation and inflammation (Additional file 1: Fig. S8A). These included: *Irf7*; interferon-stimulated genes *Isg15* and *20*; complement factor B (*Cfb*); *Axl*, a receptor tyrosine kinase that facilitates immune evasion and metastasis in various cancers [33, 34]; dual specificity phosphatases *Dusp6* and *8*; and *Map2k1*, which is frequently dysregulated in cancer and is the target of numerous experimental inhibitors being developed [35]. While the individual genes differed somewhat, this downregulation of the inflammatory gene was observed for all three treatments (Fig. 5).

However, in NBXTR3 + PLT alone, we observed something new: a marked upregulation in several innate-immune related genes—very one of which was in involved with macrophage activation (Additional file 1: Fig. S8A). These macrophage-associated genes included: *Slamf7*, a super-activator of macrophages and a strong promoter of anti-tumor phagocytosis [36]; *Abcg1*, a macrophage membrane transporter protein that mediates cholesterol efflux, promotes macrophage migration, and restrains inflammation and apoptosis [37–39]; *Lgals3*, a cell-cell adhesion model also involved in macrophage activation [40]; cathepsin S (*Ctss*), a lysosomal protease involved in peptide catalysis and antigen presentation in macrophages and DCs [41, 42]; and *Itgax*, a granulocyte integrin which promotes macrophage activation and anti-tumor immunity [43]. The protein product of *Itgax*, CD11c, also serves as the classical marker for antigen-presenting DCs [44]. Taken together, the gene expression changes within our treatment groups at the primary tumor site point to the downregulation of genetic programs involved in inflammatory and antiviral-like immunity. Moreover, when PD1, LAG3, and TIGIT were inhibited in tandem with NBTXR3-enhanced radiation, there was also a robust and unambiguous elevation of genes involved in macrophage activation, enhanced trafficking, tumor phagocytosis, and antigen presentation.

**Fig. 6:**
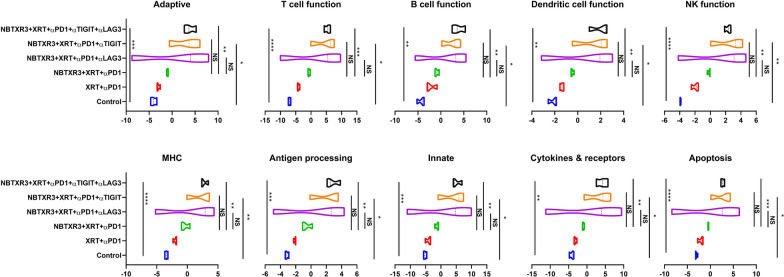
Activity score of immune pathways in the unirradiated tumors. Mice (n = 4) were treated with various combination therapies as described in Fig. 1 and were euthanized 11 d post last fraction of radiation. The RNA from the unirradiated tumor was extracted, and the immune-related genes were measured with a nCounter PanCancer Immune Profiling Panel and a nCounter MAX Analysis System. The data were analyzed with the PanCancer Immune Profiling Advanced Analysis Module. *P* < 0.05 was considered statistically significant. *P < 0.05, **P < 0.01, ***P < 0.001, NS, not significant

**Fig. 7:**
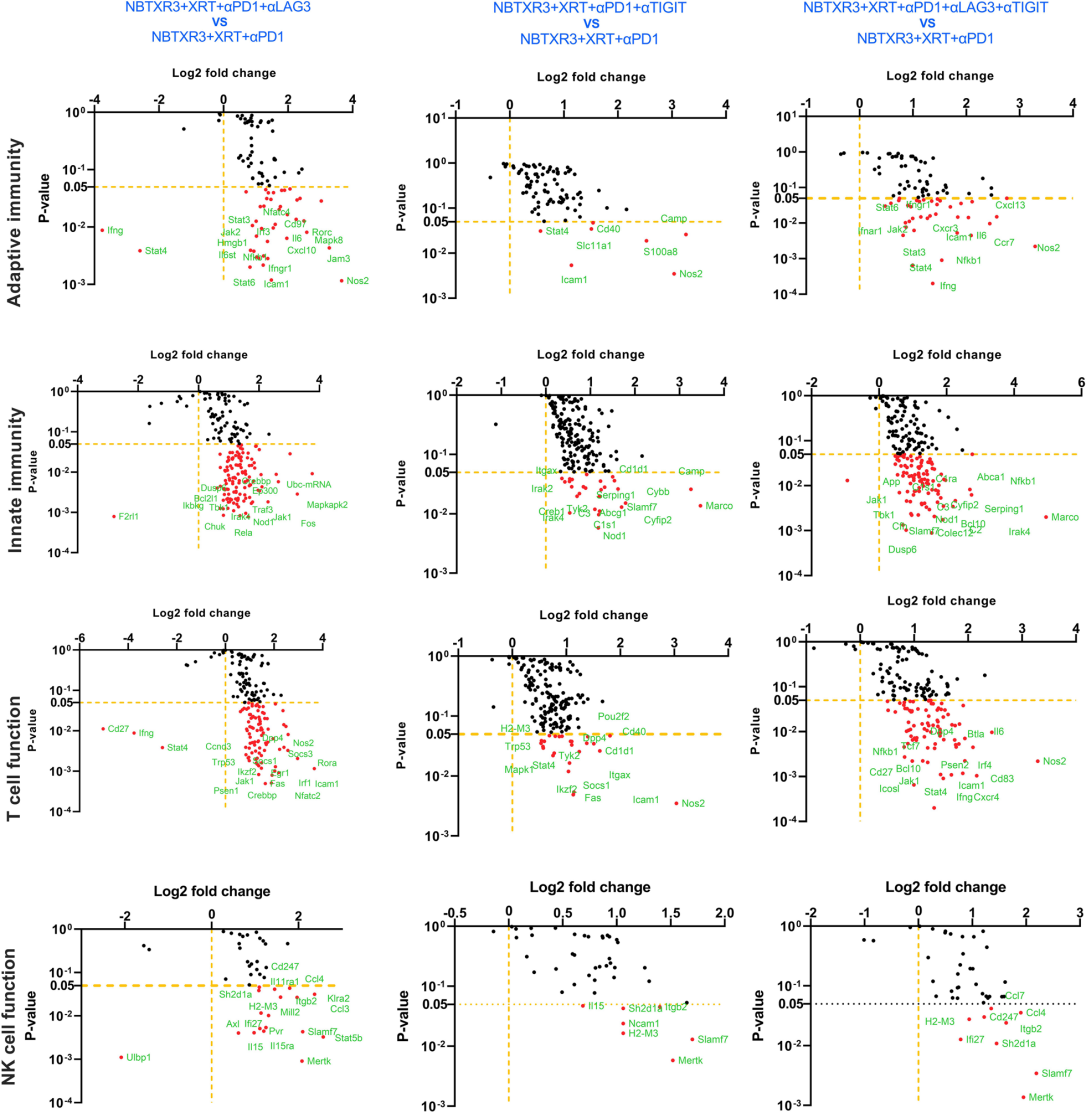
Log_2_ fold change in expression of genes involved in the adaptive pathway, innate pathway, T cell function, and NK cell function in the unirradiated tumors. Mice (n = 4) were treated with various combination therapies as described in Fig. 1 and were euthanized 11 d post last fraction of radiation. The RNA from the unirradiated tumor was extracted, and the immune-related genes were measured with a nCounter PanCancer Immune Profiling Panel and a nCounter MAX Analysis System. The data were analyzed with the PanCancer Immune Profiling Advanced Analysis Module

We next examined changes in immune-related genes in the secondary tumor (unirradiated). Unlike the primary tumor—in which only genes in pathways associated with innate immunity, antigen processing, and B cell function showed upregulation—in the secondary tumor treated with NBTXR2 + XRT + αPD + αTIGIT or αLAG3 or αLAG3 and αTIGIT, we observed marked, statistically robust increases activity and gene upregulation in all the immune pathways, including adaptive, T cell function, B cell function, dendritic cell function, innate, NK function, etc. (Figs. 6 and 7). This upregulation was the most pronounced in the mice treated with NBTXR3 + XRT + PLT (Additional file 1: Fig. S8B). Mice treated with NBXTR3 + XRT + αPD1 + αLAG3 universally experienced a broad “smear” of gene expression fold changes above and below that of untreated controls, possibly indicating highly dynamic up- and downregulation of several genes; however, most analyzed genes were upregulated. Upregulation was present—and much sharper and less ambiguous—in mice treated with NBXTR3 + XRT + αPD1 + αTIGIT. In both of these combinations (+αLAG3 and + αTIGIT), some genes were upregulated above that of the triple therapy. However, NBTXR3 + XRT + PLT boasted the “cleanest”, sharpest signal.

Given the upregulation of both innate and adaptive pathways – including pathways involved in DC function, antigen processing, T cell function, and B cell function, we suspected that what was occurring at the secondary tumor site was the recruitment of tumor-infiltrating lymphocytes (TILs) that had been primed by macrophages and DCs from the primary site. In the exploration of this hypothesis, we closely examined the individual genes that were statistically significantly (p > 0.05) upregulated in the NBTXR3 + XRT + PLT group and manually grouped them according to function (Additional file 2: Table S1). We then plotted the aggregate of the log_2_ change of each individual gene from the NBTXR3 + XRT + PLT in order to obtain a sense of which immune-related pathways were being altered following treatment (Additional file 1: Fig. S8C). Using this analysis, we found that genes involved in phagocytosis, antigen processing and presentation, cell-cell adhesion, and CD4^+^ T cell receptor (TCR) signaling were all elevated in the NBTXR3 + XRT + PLT group compared to the NBTXR3 + XRT + αPD1 group. Also upregulated were genes involved in various activating pathways associated with the immune response: the JAK-STAT pathway, MAP kinases, IRAKs and TRAFs, and NFκB, as well as various immune-associated tyrosine kinases. The expression of genes encoding numerous anti-inflammatory cytokines was also heightened. Among them was IFNγ, produced by activated CD4^+^ T cells responding to antigen recognition. In short, the genetic signature within the secondary tumor bore the unmistakable mark of robust activation of adaptive immunity through antigen presentation.

Also present was the genetic signature of a vigorous innate immune response. As previously mentioned, genes governing phagocytosis—the process whereby innate immune cells, mostly macrophages, engulf target cells—were highly upregulated. So too were genes involved in reactive oxygen species (ROS) generation, typically upregulated by activated innate immune cells to further their activation and digesting their prey engulfed through phagocytosis. Several genes involved in the complement system, a central mediator of radiotherapy-induced tumor-specific immunity [45], were also upregulated. Among the most highly upregulated gene groupings were those specifically associated with macrophage identity and function. The gene for macrophage colony-stimulating factor (*Csf1*) and its receptor, *Csf1r*, were both elevated, as were the genes for natural resistance-associated macrophage protein 1 (*Slc11a1*), macrophage receptor with collagenous structure (*Marco*), and macrophage inflammatory protein 1 β (*Ccl4*). We, moreover, observed an even greater upregulation of the macrophage super-activator *Slamf7*. Altogether, our NanoString data paint a picture of strong innate and adaptive immune responses at the secondary tumor site.

### NBTXR3 + XRT + PLT treatment produces long-term immunological memory

As demonstrated in the results above, antitumor immune response plays a vital role in the NBTXR3 + XRT + PLT therapy resulted tumor eradication. The most robust immune responses are marked by the development of immunological memory, in which a small remnant of antigen-specific T cells and B cells activated in the initial antigen exposure persist, primed, and ready to respond rapidly should the organism ever be challenged by the same pathogen. The abscopal effect is thought to stimulate such a response, essentially converting the primary tumor into an in situ vaccine [46].

**Fig. 8:**
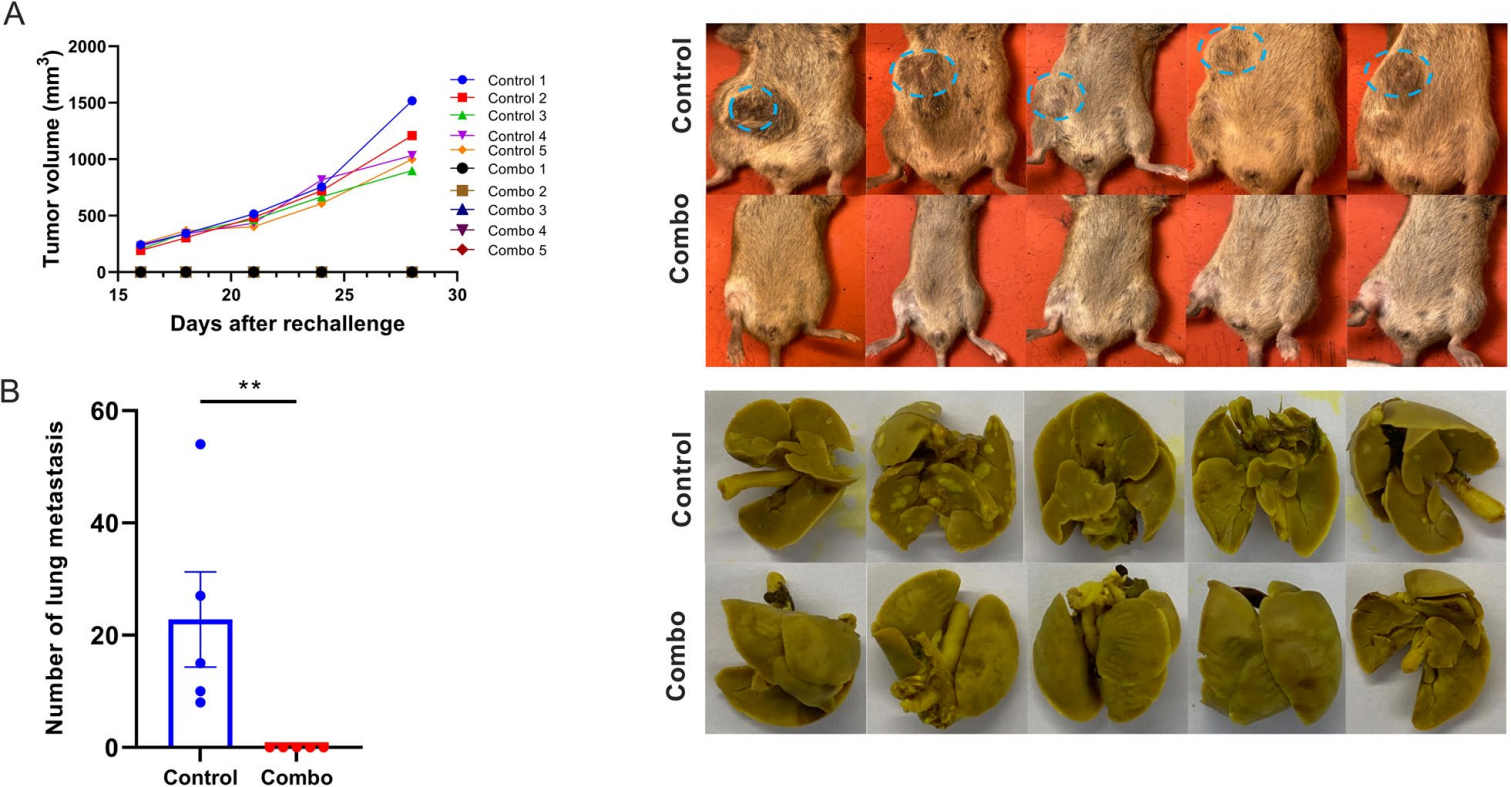
NBTXR3 + XRT + PLT (Combo) treated mice reject tumor re-challenge. **A** Tumor volume and images of the tumors in the mice. **B** The number of lung metastases and images of the lung tumors. The five mice that survived in the NBTXR3 + XRT + PLT group in Fig. 1 were re-challenged with 5 × 10^4^ 344SQR cells on the right flank at least 60 d after the last fraction of 12 Gy. Five mice with 5 × 10^4^ 344SQR cells inoculated on the right flank served as the control. All the mice were euthanized 28 d after tumor inoculation. Lungs were harvested from the mice, and the number of lung metastases was counted. *P* < 0.05 was considered statistically significant. **P < 0.01, NS, not significant

To evaluate if the cured mice developed an antitumor memory immune response, the 5 survivor mice from the NBTXR3 + XRT + PLT group were re-injected with 5 × 10^4^ 344SQR cells on the right flank, and their tumor growth was monitored. None of these mice developed tumors (Fig. 8A). In contrast, all mice in the control group did. Twenty-eight days post tumor re-challenge, when the mice in the control group had reached the experimental endpoint, all mice were sacrificed, and their lung metastases were counted. No lung metastasis was observed in the NBTXR3 + XRT + PLT group (Fig. 8B); however, all of the mice in the control group developed various numbers of tumor nodules in their lungs.

**Fig. 9:**
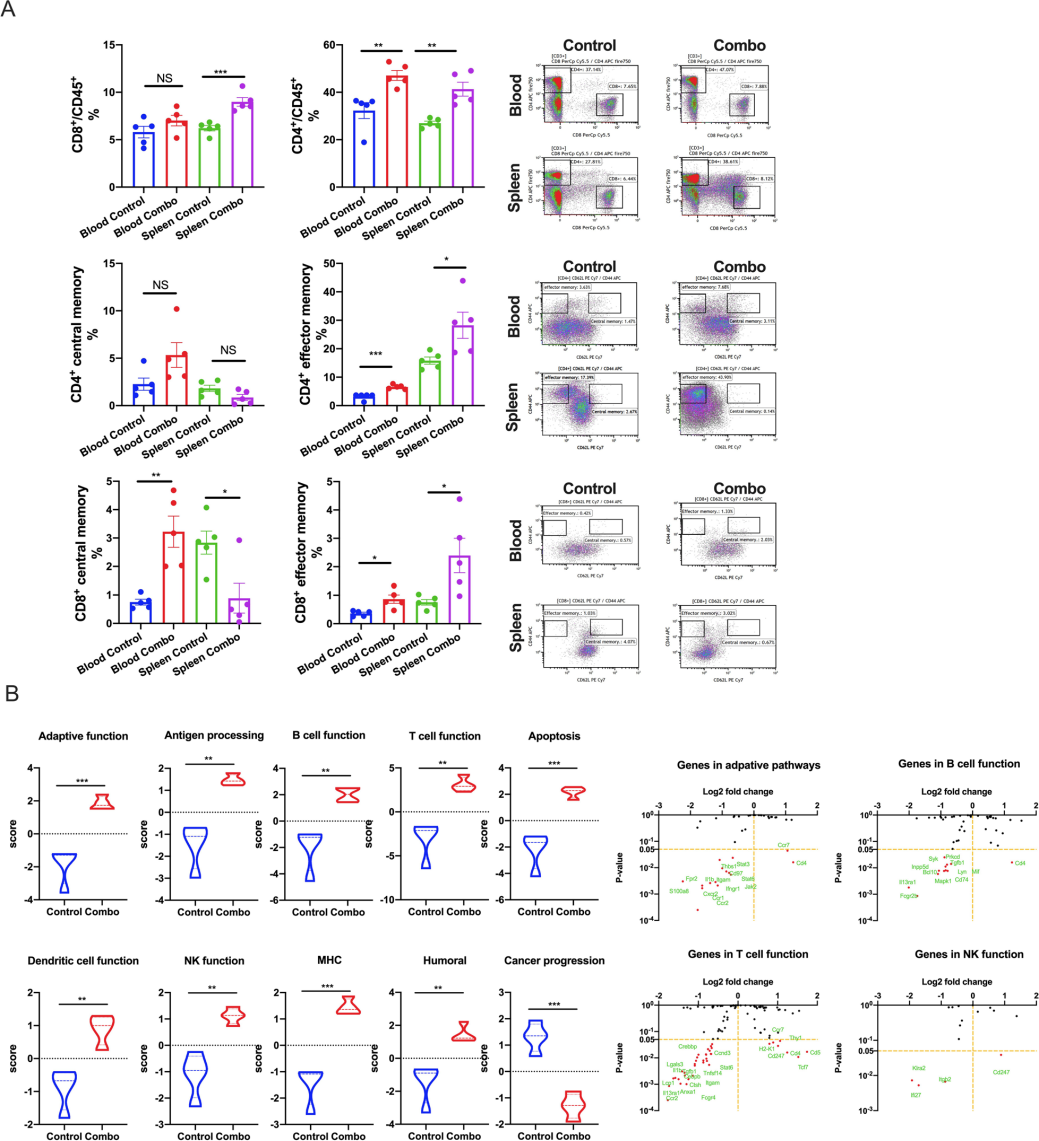
NBTXR3 + XRT + PLT (Combo) induces immunological memory and upregulates immune activities in the blood. **A** Percentages of CD4^+^/CD45^+^ and CD8^+^/CD45^+^ T cells and memory CD4^+^ and CD8^+^ T cells in the blood and spleen. **B** Nanostring activity score of immune pathways and log_2_ fold change in expression of genes involved in the adaptive pathway, NK cell function, B cell function, and T cell function with the control as the baseline. The five mice that survived in the NBTXR3 + XRT + PLT group in Fig. 1 were re-challenged with 5 × 10^4^ 344SQR cells on the right flank at least 60 d after the last fraction of 12 Gy. Five mice with 5 × 10^4^ 344SQR cells inoculated on the right flank served as the control. All the mice were euthanized 28 d after tumor inoculation. Blood was harvested before euthanizing the mice, red blood cells were lysed, and RNA was extracted for Nanostring analysis. The populations of memory CD4^+^ and CD8^+^ T cells were analyzed by flow cytometry. *P* < 0.05 was considered statistically significant. *P < 0.05, **P < 0.01, ***P < 0.001, NS, not significant

We next looked directly at the levels of total, central, and effector memory T cells (Fig. 9A). We collected blood and spleens from both experimental groups, from which we isolated CD4^+^ and CD8^+^ memory T cells. The differences in memory composition and distribution were broadly similar between both T cell subsets. For both CD4^+^ and CD8^+^ cells, total (CD45^+^) and effector memory T cells (T_EM_ cells; CD44^+^CD62L^-^) were elevated within the blood and spleens of NBTXR3 + XRT + PLT-treated mice (though this elevation was not statistically significant for blood total memory CD8^+^ T cells). Central memory T cells (T_CM_ cells; CD44^+^CD62L^+^) of both compartments were enriched in the blood – significantly so in the case of CD8^+^ T cells; curiously, however, T_CM_ cells of both compartments were less abundant within the spleens of treated mice than controls (again, this was statistically significant for CD8^+^ T cells, not so for CD4^+^). Given the context of this observation (i.e., during an ongoing immune challenge), we suspect that these numbers paint the picture of a poised memory response that has been sprung, triggering a mass re-activation of T_CM_ cells of both subsets, their differentiation into T_EM_ cells [47], and their exodus from the spleen into the bloodstream.

Finally, we examined peripheral blood monocytes (PBMCs) isolated for gene upregulation using the same Nanostring module previously described. PBMCs from mice treated with NBTXR3 + XRT + PLT exhibited significant upregulation in every immune-related genetic program examined, and a significant downregulation of genes associated with cancer progression (Fig. 9B). This, in coordination with the lack of any tumor development and the hardy memory T cell response, indicates that mice treated with NBTXR3 + XRT + PLT were not only able to survive and clear the initial tumor challenge, but were able to develop immunological memory that inoculated them against the same tumor type.

## Discussion

Radiotherapy, traditionally used for localized tumor treatment, is increasingly combined with CPIs to treat metastasized tumors [48, 49]. Radiation can kill cancer cells, release tumor antigens, and activate tumor-specific immune responses [50]. Consequently, tremendous effort has been made to optimize radiotherapy to maximize its immune-activation capacity [51, 52].

In pursuit of this end, we previously discovered that the radiation-enhancing nanoparticle NBTXR3 could significantly promote the activation of radiotherapy-mediated antitumor immunity. However, the activated effector immune cells, including CD4^+^ T cells, CD8^+^ T cells, and NK cells, are subject to exhaustion [53]. Numerous ICRs, such as PD1, LAG3, and TIGIT, have been found to participate in the exhaustion of immune cells [54]. Therefore, in this study, we explored the possibility of increasing immune activation while reducing immune cell exhaustion through combining NBTXR3-enhanced radiation with blockade of LAG3, TIGIT, and PD1.

In agreement with our previous findings [20], mice treated with NBTXR3 + XRT + αPD1 displayed improved control of both primary and secondary tumors over XRT + αPD1. No treatment group that lacked NBTXR3 showed a robust therapeutic response, even with triple CPI, alone or with radiation. These results demonstrate the impressive potency of NBTXR3 in amplifying tumor clearance. We found, moreover, that adding αLAG3 or αTIGIT alone to NBTXR3 + XRT + αPD1 significantly augmented this anti-tumor effect, decreasing the growth of both tumors in a manner superior to that of NBTXR3 + XRT + αPD1 without further CPI. Furthermore, inhibiting all three checkpoints simultaneously, along with NBTXR3-amplified irradiation (NBTXR3 + XRT + PLT), achieved unparalleled efficacy in tumor clearance, reduction of metastasis, induction of immunological memory, and overall survival. Indeed, the survival rate of mice treated with NBTXR3 + XRT + PLT was 37.5%, compared to 0% survival in the other groups.

There have been reports showing that the dual immunotherapies of αLAG3 + αPD1 or αTIGIT + αPD1 can effectively enhance antitumor immunity and inhibit tumor growth in other tumor models [55, 56]. However, the results of this study suggest that in tumors already resistant to αPD1, NBTXR3-enhanced radiation is essential for this CPI-mediated immune response. We hypothesize that this is likely due to the initial radiation-induced trauma to the primary tumor causing inflammation and tumor-associated antigen exposure, effectively penetrating the immune privilege enjoyed by the tumor and kick-starting immune activation. The corollary of this hypothesis is that, just as immune activation requires NBTXR3-amplified XRT to initiate, NBTXR3-amplified XRT requires immune activation to potentiate the full therapeutic effect of the treatment. Indeed, we found that control of tumor growth and animal survival were ablated when immune cells were selectively depleted.

Tumor clearance is typically considered the domain of cytolytic immune cell populations—CD8^+^ T cells and natural killer (NK) T cells. However, the immune population whose depletion most strongly impacted the therapy’s efficacy were CD4^+^ T cells, the so-called helper T cells. Helper T cells serve as the generals of the immune response, directing and selectively enhancing the activities of other immune cells through the secretion of cytokines. Like generals, CD4^+^ T cells require information from scouts on the nature of the threat. In the immune system, this information comes to the CD4^+^ T cells in the form of tumor antigenic peptides presented by antigen-presenting cells (APCs), the most prominent members of which are dendritic cells (DCs) and macrophages. Macrophages are typically first on the scene of a site of inflammatory injuries – such as from radiation damage to a tumor. Once there, they set to work engulfing damaged and pathogenic cells through phagocytosis, digesting the engulfed cells, and presenting the digested peptides to CD4^+^ T cells *via* class II major histocompatibility complexes (MHCs). The CD4^+^ T cells, having thus been appraised of the threat, secrete inflammatory cytokines such as IFNγ, stimulating the macrophages to step up their attack. They, in turn, begin secreting their own inflammatory cytokines such as IL-6 and tumor necrosis factor (TNF), producing caustic reactive oxygen species (ROSs) that seep into the tumor and induce apoptosis, and intensifying their phagocytic engulfment of diseased or damaged cells. CD4^+^ have been previously observed to be the primary mediators of tumor clearance in a manner that is independent of CD8^+^ T cells but reliant on IFNγ [57], and the partnership between CD4^+^ T cells and macrophages has been documented as one of the mechanisms driving this effect [58].

When we looked at the mRNA transcriptomic signature of primary and secondary tumors of mice treated with NBTXR3 + XRT + PLT, we observed a textbook upregulation of genes participating in every step of this process. At the primary tumor, we saw strong upregulation of genes primarily associated with macrophage activation. When we turned to the secondary tumor, we saw a full-scaled immune response activation. Genes associated with antigen presentation were upregulated, as were genes associated with macrophage function and identity. In accordance with this, numerous genes specifically involved in phagocytosis and ROS generation were also heightened.

Furthermore, we detected the upregulation of genes associated with T cell activation—specifically, CD4^+^ T cell activation. Inflammatory cytokines such as IFNγ and IL-6, components of their receptors, and downstream signaling intermediaries were likewise elevated. Several members of the complement system, which can be generated by both DCs and T cells, were also increased. While classically considered antibacterial and antiparasitic defense, the complement system can also be activated by the altered membranes of tumor cells induced into apoptotic and necrotic cells [45]. Conspicuously absent were any genes associated with CD8^+^ T cell-specific responses.

Immune-related gene upregulation in the secondary tumor was much weaker in mice treated with NBTXR3 + XRT + αPD1 and αTIGIT than in mice treated with NBTXR3 + XRT + PLT. However, it was equally robust in mice treated with NBTXR3 + XRT + αPD1 and αLAG3 as in NBTXR3 + XRT + PLT-treated mice. While control of primary tumor growth and overall survival was greater for NBTXR3 + XRT + PLT-treated mice than for mice treated with only NBTXR3 + XRT + αPD1 and αLAG3, control of the secondary tumor growth was roughly equivalent between them. These results suggest that the key difference in outcome between these two treatments stems from controlling the primary tumor. Another possibility, not exclusive to the previous, stems from the same genes upregulated in the secondary tumors of mice treated with NBTXR3 + XRT + PLT versus those treated with NBTXR3 + XRT + αPD1 and αLAG3. In particular, we found that while the gene for IFNγ was, in fact, downregulated in mice treated with NBTXR3 + XRT + αPD1 and αLAG3 (as compared to mice treated with only NBTXR3 + XRT + αPD1), it was markedly upregulated in mice treated with NBTXR3 + XRT + PLT. Also of note is that in mice those treated with NBTXR3 + XRT + αPD1 and αLAG3, the gene for PD-L1 was expressed at a level higher than that expressed by mice treated with NBTXR3 + XRT + PLT, possibly indicating greater immune exhaustion.

Regardless of the cause, what is clear is that the differential combination of αPD1, αLAG3, and αTIGIT in combination with NBTXR3-amplified irradiation achieves similar but distinct outcomes. We suspect that what might underlie the differences between these therapies is that the combination of NBTXR3-enhanced irradiation of the primary tumor, in tandem with blockade of all three ICRs, serves to push the immune response over some threshold at the primary tumor site, allowing complete or nearly complete clearance thereof. This effect is not attributable to the action of any of these components, as only the presence of all of them together achieves this outcome. How exactly the elements of this combined approach harmonize in such a way as to achieve this highly favorable outcome is richly deserving of further study. What is clear, however, is that the key lies in how each lifted checkpoint influences the immune response.

The heavy dependence of the NBTXR3 + XRT + PLT therapy on the immune responses is reflected by the faster growth of both the primary and the secondary tumors after depletion of CD4^+^ T cells, CD8^+^ T cells, and NK cells. It is also noteworthy that these three types of immune populations have varied influences on antitumor efficacy, in which depletion of CD4^+^ T cells completely abrogated the antitumor effect. In contrast, depletion of NK cells had the least impact on the antitumor activity. Other studies have also reported results that attest to the indispensability of CD4^+^ T cells to the efficacy of IRT [59, 60]. The critical role of CD4^+^ T cells may be attributed to the fact that they lie upstream of CD8^+^ T cell- and NK cell-mediated antitumor pathways.

The reliance of this combinatorial therapy on the immune system—and its potential for stimulating it—is perhaps no better illustrated than with the strong memory response to repeat exposure to the same tumor cells in mice that cleared the initial tumors. These mice display superior memory cell numbers and mobility to their uninoculated counterparts, effectively immunizing them against relapse, even when it is deliberately induced. This remarkable finding indicates that this line of therapy may provide patients with a highly favorable response and even complete and enduring remission.

## Conclusion

In conclusion, this study demonstrates that the combination therapy of NBTXR3 + XRT + αPD1 significantly increases the expression of TIGIT and LAG3 in both irradiated and unirradiated tumors. Adding αLAG3 and αTIGIT to NBTXR3 + XRT + αPD1 enables significant improvement in the control of both primary and secondary tumors. This combination therapy facilitates the infiltration of immune cells into the tumors, increases the proliferation of CD4^+^ and CD8^+^ T cells, and enhances the activities of a wide range of immune pathways. In addition, it produces a long-term and potent memory immune response against cancer cells and effectively prevents the re-development of tumors. These data attest to the profound potential of combining checkpoint inhibition with radiotherapy and provide the rationale for further exploration of this line of therapy.

## Supplementary Information

**Additional file 1: Figure S1.** Log_2_ fold change in the expression of TIGIT and LAG3 in tumors treated with NBTXR3 + XRT + αPD1. The 344SQR cells (5 × 10^4^) were subcutaneously injected into the right legs of the 129/SvEv syngeneic female mice (n = 4, 8–12 weeks old) on day 0 to establish the “primary” tumor and into the left legs on day 4 to establish the “secondary” tumor. The primary tumors were intratumorally injected with NBTXR3 on day 7, followed by three fractions of 12 Gy radiation on days 8, 9, and 10. αPD1 (200 µg) was intraperitoneally injected into the mice on days 5, 8, 11, and 14. The expression of LAG3 and TIGIT in the irradiated and unirradiated tumors harvested on day 21 were measured by Nanostring. The mice inoculated with both primary and secondary tumors but did not receive any treatment served as control. *P* < 0.05 was considered statistically significant. **P < 0.01, ***P < 0.001. **Figure S2.** Individual tumor growth curves in mice receiving treatment as indicated in Fig. 1.**Figure S3.** Blockade of LAG3 and TIGIT with the combination of NBTXR3 + XRT + αPD1 reduces the number of lung metastases. The mice (n = 5) were treated with various combination therapies, as illustrated in Fig. 1, and were euthanized on day 21. The lungs were harvested and stored in Bouin’s fixative solution for three days, after which metastatic nodules were counted. The number of lung metastases was compared by t-test and were expressed as mean ± standard error of the mean (SEM). *P* < 0.05 was considered statistically significant. *P < 0.05, **P < 0.01, ***P < 0.001, ****P < 0.0001, NS, not significant. **Figure S4.** Body weight of mice (n = 5) treated with combination therapy of NBTXR3 + XRT + αPD1 + αLAG3 + αTIGIT as shown ing Fig. 1A. **Figure S5.** Dual blockade of TIGIT and LAG3 promotes proliferation of CD4^+^ and CD8^+^ T cells. **(A)** Representative flow cytometry images of Ki67^+^CD4^+^ and Ki67^+^CD8^+^ T in primary tumors. **(B)** Representative flow cytometry images of Ki67^+^CD4^+^ and Ki67^+^CD8^+^ T cells in secondary tumors. **(C)** Representative flow cytometry images of Ki67^+^CD4^+^ and Ki67^+^CD8^+^ T cells in the blood. The mice (n = 5) were treated with various combination therapies, including XRT + αPD1, NBTXR3 + XRT + αPD1, NBTXR3 + XRT + αPD1 + αLAG3, NBTXR3 + XRT + αPD1 + αTIGIT, and NBTXR3 + XRT + PLT as indicated in Fig. 1A and were sacrificed on day 21. The mice which were inoculated with tumors only served as control. Immune cells from primary tumors, secondary tumors, and blood were processed and stained with αCD45-APC-Cy7, αCD3-PE-Cy7, αCD4-alexa 700, αCD8-PercpCy5.5, and αKi67-alexa 647. The cells were run with a Gallios Flow Cytometer (Beckman Coulter) and analyzed with Kaluza software Version 2.1. **Figure S6.** Flow cytometry gating strategy in Fig. 3. **Figure S7.** Nanostring cell score of various immune cells treated with different combinations of NBTXR3, XRT, αPD1, αLAG3 and αTIGIT in both primary and secondary tumors. Mice (n = 4) were treated with various combination therapies as described in Fig. 1 and were euthanized 11 d post last fraction of radiation. The RNA from the irradiated and unirradiated tumors was extracted, and the immune-related genes were measured with a nCounter PanCancer Immune Profiling Panel and a nCounter MAX Analysis System. The data were analyzed with the PanCancer Immune Profiling Advanced Analysis Module. The cell scores were compared by t-test and were expressed as mean ± standard error of the mean (SEM). P < 0.05 was considered statistically significant. ***P < 0.001, NS, not significant. **Figure S8.** Multiple immune-related genes are significantly differentially expressed in the tumors of mice following supplementation of NBTXR3 + XRT + αPD1 with αLAG3, αTIGIT, or both. Statistical significance was assessed *via a* two-tailed t-test, with a p < 0.05. **A** Heatmap of genes significantly up- or down-regulated in at least one of the three treatment groups relative to NBTXR3 + XRT + αPD-1 in the primary tumor. **B** Heatmap of genes significantly up- or down-regulated in at least one of the three treatment groups relative to NBTXR3 + XRT + αPD1 in the secondary tumor. Two hundred and forty-six (246) such genes were identified and are here ranked alphabetically. **C** Genes identified as significantly upregulated in NBTXR3 + XRT + PLT-treated mice compared to NBTXR3 + XRT + αPD1-treated mice were manually grouped according to their established function. Functional groups were plotted according to the log_2_ fold change from baseline (NBTXR3 + XRT + αPD1) of their constituent genes, represented in aggregate *via* violin plot.

**Additional file 2: Table S1.** Log2 fold change and the function of the genes which were significantly up-regulated in the unirradiated tumors treated with NBTXR3+XRT+PLT vs. NBTXR3+XRT+αPD1.

## Data Availability

The data and materials supporting this study’s findings are available from the corresponding author upon reasonable request.
