# Supplementary material for: Combining a nanoparticle-mediated immunoradiotherapy with dual blockade of LAG3 and TIGIT improves the treatment efficacy in anti-PD1 resistant lung cancer
Source: J Nanobiotechnology. 2022 Sep 19;20:417. doi: 10.1186/s12951-022-01621-4 (PMC9484155; doi:10.1186/s12951-022-01621-4)
Supplement: Supplementary file 1 — Additional file 1: Figure S1. Log2 fold change in the expression of TIGIT and LAG3 in tumors treated with NBTXR3 + XRT + αPD1. The 344SQR cells (5 × 10^4^) were subcutaneously injected into the right legs of the 129/SvEv syngeneic female mice (n = 4, 8–12 weeks old) on day 0 to establish the “primary” tumor and into the left legs on day 4 to establish the “secondary” tumor. The primary tumors were intratumorally injected with NBTXR3 on day 7, followed by three fractions of 12 Gy radiation on days 8, 9, and 10. αPD1 (200 µg) was intraperitoneally injected into the mice on days 5, 8, 11, and 14. The expression of LAG3 and TIGIT in the irradiated and unirradiated tumors harvested on day 21 were measured by Nanostring. The mice inoculated with both primary and secondary tumors but did not receive any treatment served as control. P < 0.05 was considered statistically significant. **P < 0.01, ***P < 0.001. Figure S2. Individual tumor growth curves in mice receiving treatment as indicated in Fig. 1.Figure S3. Blockade of LAG3 and TIGIT with the combination of NBTXR3 + XRT + αPD1 reduces the number of lung metastases. The mice (n = 5) were treated with various combination therapies, as illustrated in Fig. 1, and were euthanized on day 21. The lungs were harvested and stored in Bouin’s fixative solution for three days, after which metastatic nodules were counted. The number of lung metastases was compared by t-test and were expressed as mean ± standard error of the mean (SEM). P < 0.05 was considered statistically significant. *P < 0.05, **P < 0.01, ***P < 0.001, ****P < 0.0001, NS, not significant. Figure S4. Body weight of mice (n = 5) treated with combination therapy of NBTXR3 + XRT + αPD1 + αLAG3 + αTIGIT as shown ing Fig. 1A. Figure S5. Dual blockade of TIGIT and LAG3 promotes proliferation of CD4+ and CD8+ T cells. (A) Representative flow cytometry images of Ki67+CD4+ and Ki67+CD8+ T in primary tumors. (B) Representative flow cytometry images of Ki67+CD4+ and Ki67+CD8+ T [file 12951_2022_1621_MOESM1_ESM.docx]

**Supplemental materials**

**
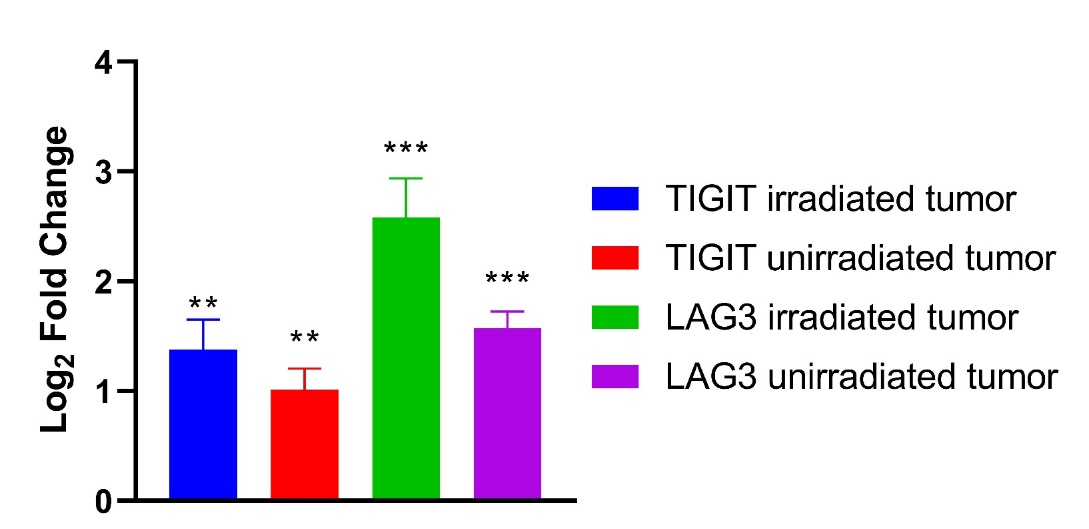
**

**Fig. S1** Log_2_ fold change in the expression of TIGIT and LAG3 in tumors treated with NBTXR3+XRT+αPD1. The 344SQR cells (5x10^4^) were subcutaneously injected into the right legs of the 129/SvEv syngeneic female mice (n=4, 8-12 weeks old) on day 0 to establish the “primary” tumor and into the left legs on day 4 to establish the “secondary” tumor. The primary tumors were intratumorally injected with NBTXR3 on day 7, followed by three fractions of 12 Gy radiation on days 8, 9, and 10. αPD1 (200 µg) was intraperitoneally injected into the mice on days 5, 8, 11, and 14. The expression of LAG3 and TIGIT in the irradiated and unirradiated tumors harvested on day 21 were measured by Nanostring. The mice inoculated with both primary and secondary tumors but did not receive any treatment served as control. *P*<0.05 was considered statistically significant. ***P*<0.01, ****P*<0.001.


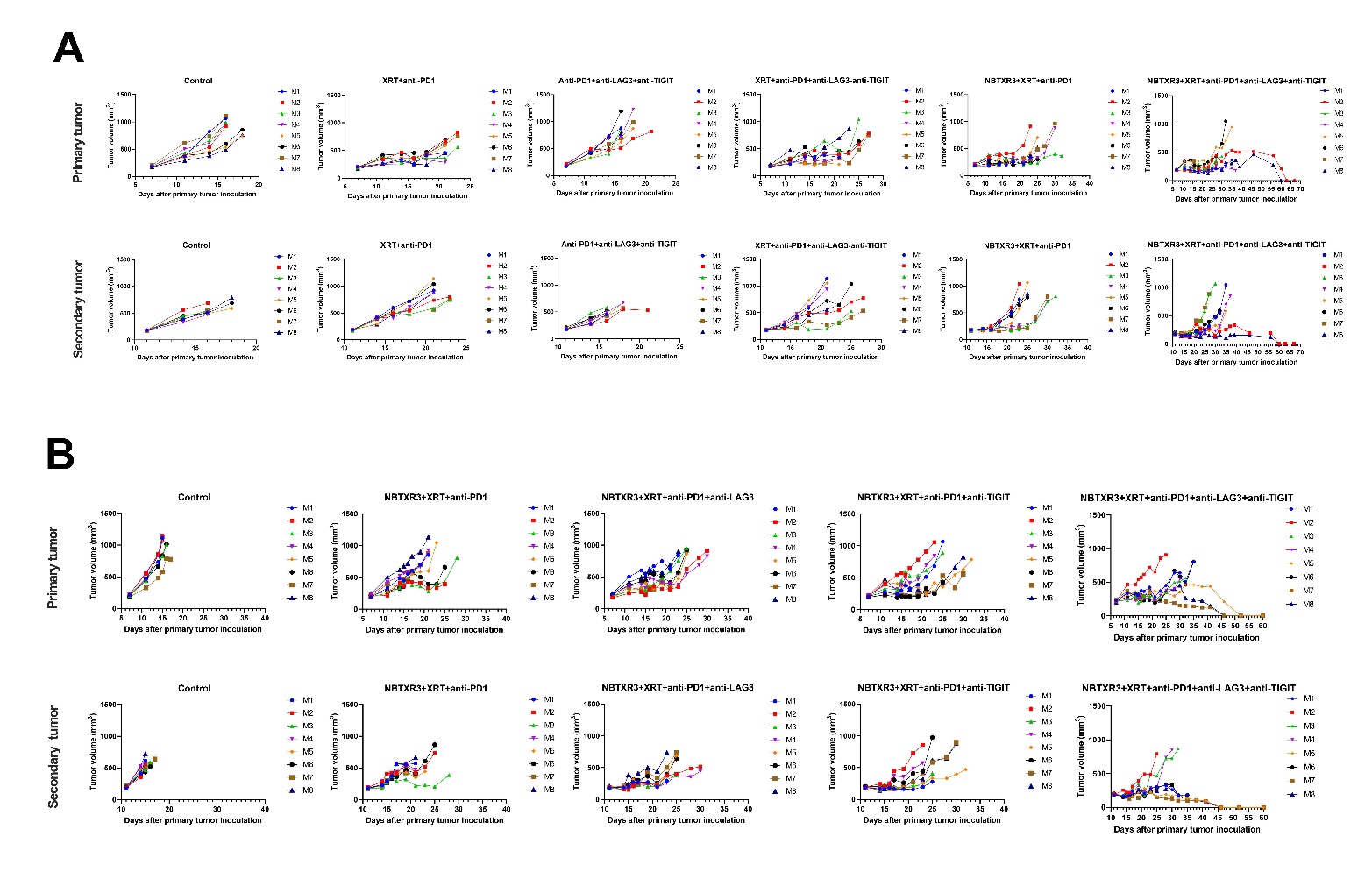


**Fig. S2** Individual tumor growth curves in mice receiving treatment as indicated in **Fig. 1**.


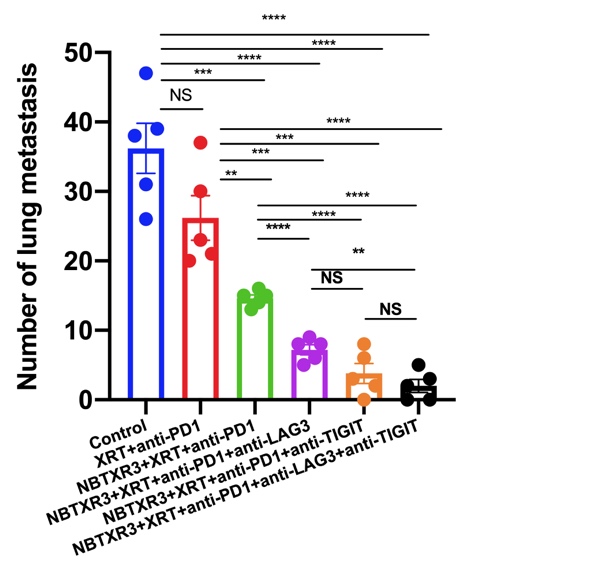


**Fig. S3** Blockade of LAG3 and TIGIT with the combination of NBTXR3+XRT+αPD1 reduces the number of lung metastases. The mice (n=5) were treated with various combination therapies, as illustrated in **Fig. 1**, and were euthanized on day 21. The lungs were harvested and stored in Bouin’s fixative solution for three days, after which metastatic nodules were counted. The number of lung metastases was compared by t-test and were expressed as mean ± standard error of the mean (SEM). *P*<0.05 was considered statistically significant. **P*<0.05, ***P*<0.01, ****P*<0.001, *****P*<0.0001, NS, not significant.


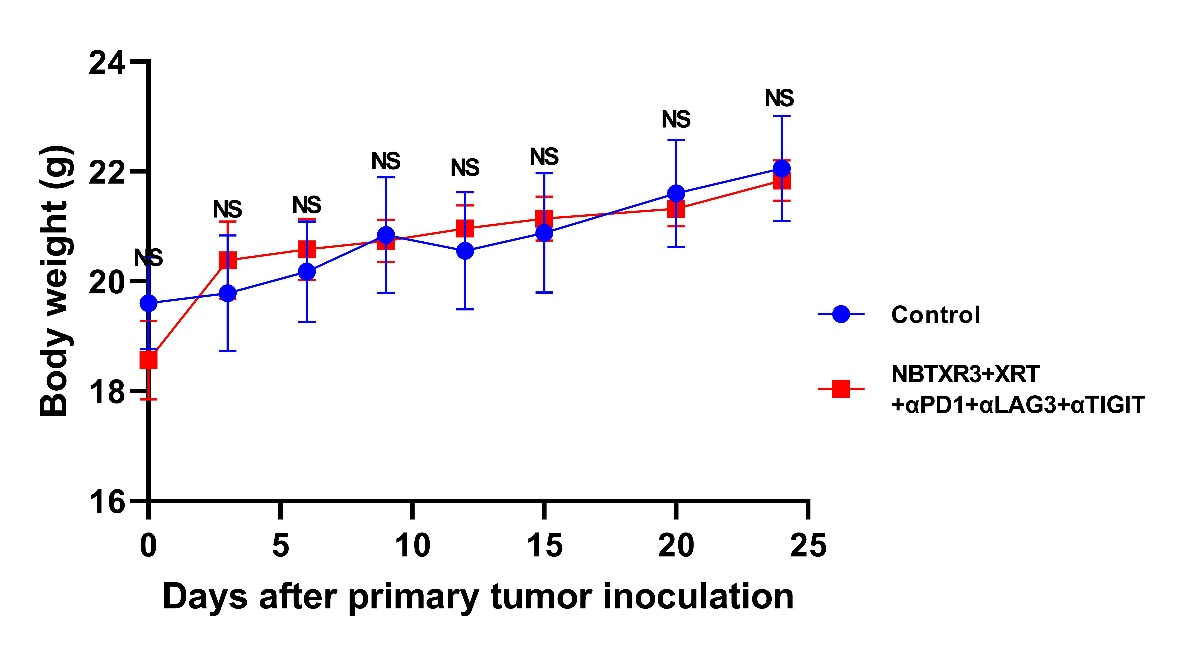


**Fig. S4** Body weight of mice (n=5) treated with combination therapy of NBTXR3+XRT+αPD1+αLAG3+αTIGIT as shown in Figure 1A.


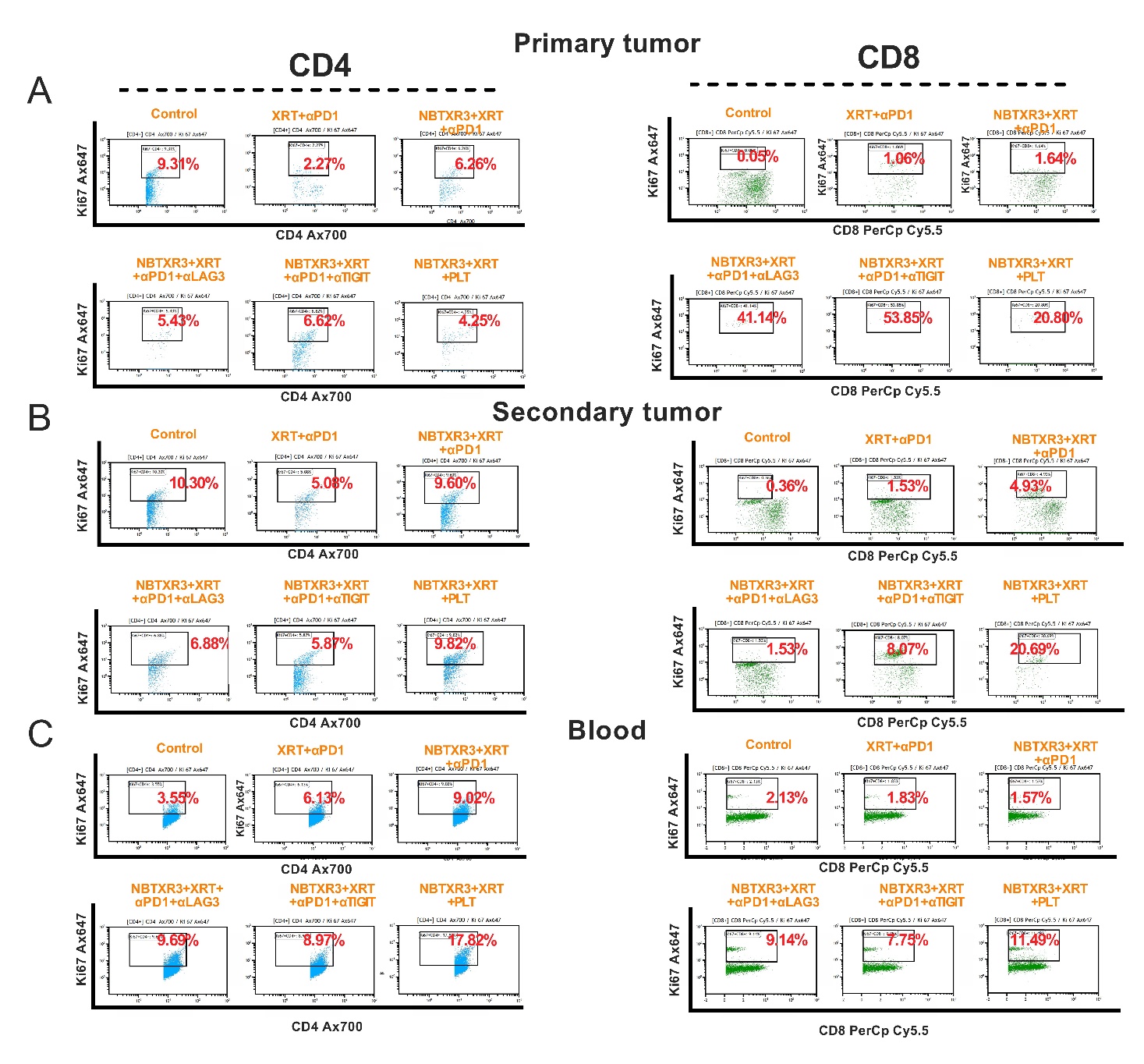


**Fig. S5** Dual blockade of TIGIT and LAG3 promotes proliferation of CD4^+^ and CD8^+^ T cells. **(A)** Representative flow cytometry images of Ki67^+^CD4^+^ and Ki67^+^CD8^+^ T in primary tumors. **(B)** Representative flow cytometry images of Ki67^+^CD4^+^ and Ki67^+^CD8^+^ T cells in secondary tumors. **(C)** Representative flow cytometry images of Ki67^+^CD4^+^ and Ki67^+^CD8^+^ T cells in the blood. The mice (n=5) were treated with various combination therapies, including XRT+αPD1, NBTXR3+XRT+αPD1, NBTXR3+XRT+αPD1+αLAG3, NBTXR3+XRT+αPD1+αTIGIT, and NBTXR3+XRT+PLT as indicated in **Fig. 1A** and were sacrificed on day 21. The mice which were inoculated with tumors only served as control. Immune cells from primary tumors, secondary tumors, and blood were processed and stained with αCD45-APC-Cy7, αCD3-PE-Cy7, αCD4-alexa 700, αCD8-PercpCy5.5, and αKi67-alexa 647. The cells were run with a Gallios Flow Cytometer (Beckman Coulter) and analyzed with Kaluza software Version 2.1.


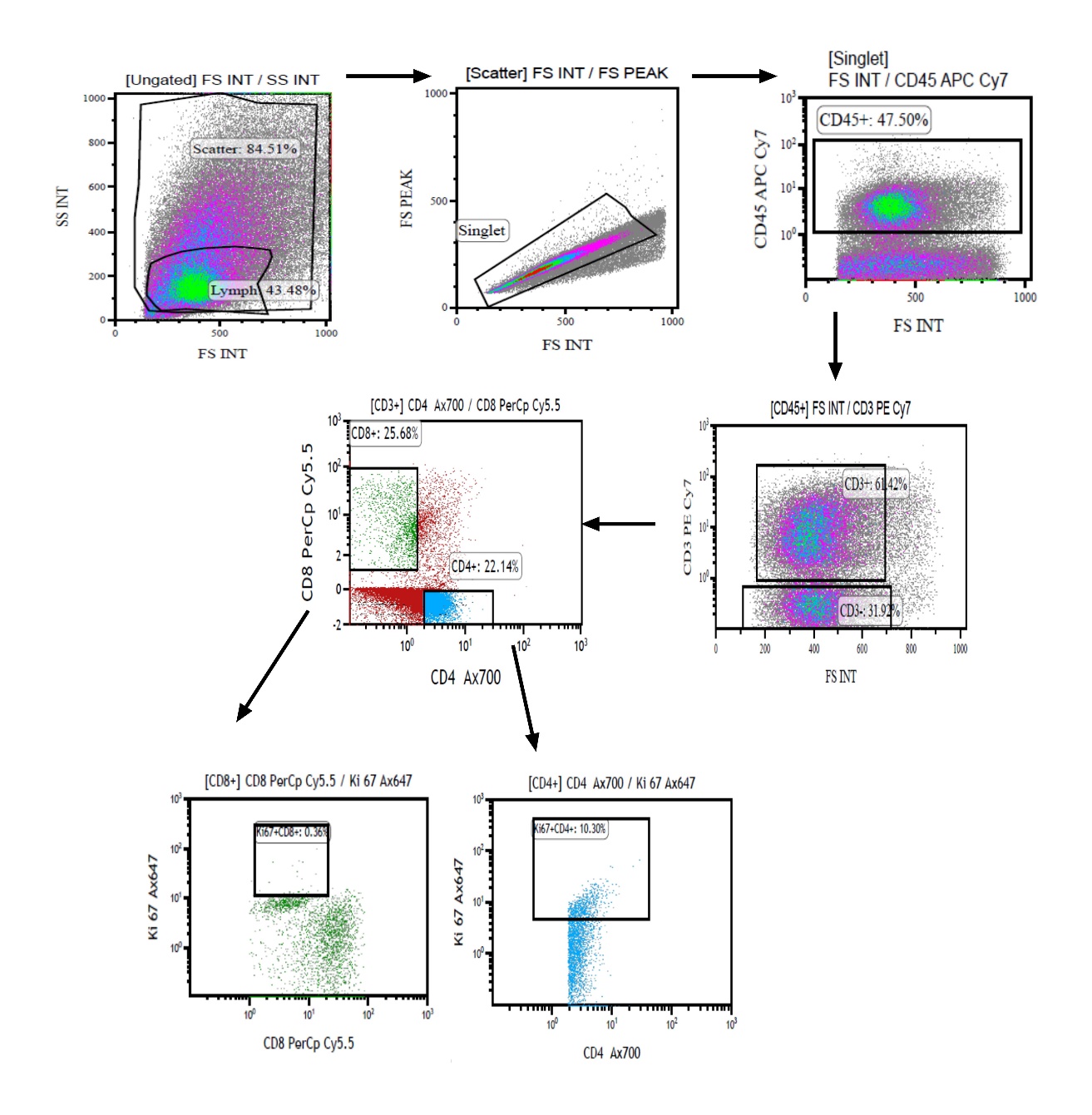


**Fig. S6** Flow cytometry gating strategy in **Fig. 3**.


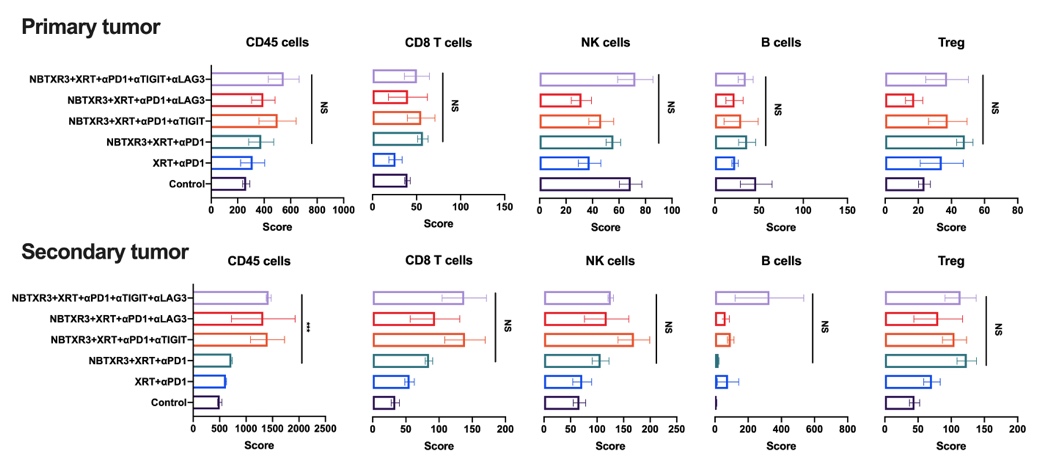


**Fig. S7** Nanostring cell score of various immune cells treated with different combinations of NBTXR3, XRT, αPD1, αLAG3 and αTIGIT in both primary and secondary tumors. Mice (n=4) were treated with various combination therapies as described in **Fig. 1** and were euthanized 11 d post last fraction of radiation. The RNA from the irradiated and unirradiated tumors was extracted, and the immune-related genes were measured with a nCounter PanCancer Immune Profiling Panel and a nCounter MAX Analysis System. The data were analyzed with the PanCancer Immune Profiling Advanced Analysis Module. The cell scores were compared by t-test and were expressed as mean ± standard error of the mean (SEM). P<0.05 was considered statistically significant. ***P<0.001, NS, not significant.


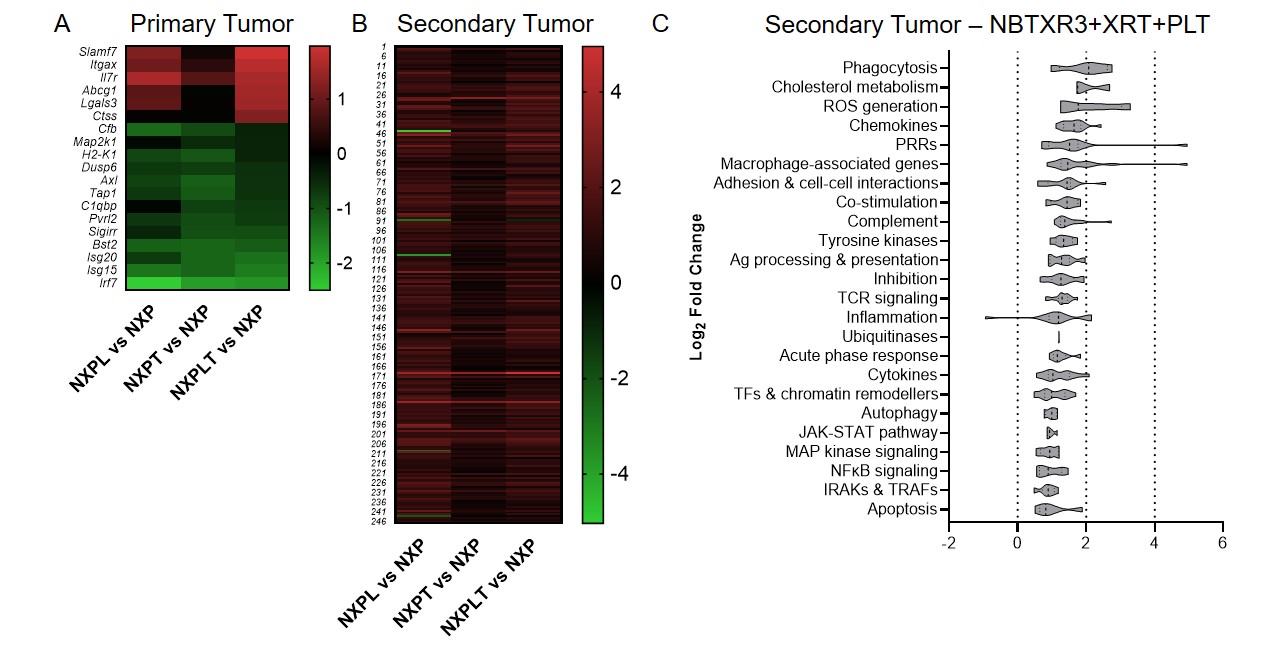


**Fig. S8** Multiple immune-related genes are significantly differentially expressed in the tumors of mice following supplementation of NBTXR3+XRT+αPD1 with αLAG3, αTIGIT, or both. Statistical significance was assessed *via a* two-tailed t-test, with a p<0.05. **(A)** Heatmap of genes significantly up- or down-regulated in at least one of the three treatment groups relative to NBTXR3+XRT+αPD-1 in the primary tumor. **(B)** Heatmap of genes significantly up- or down-regulated in at least one of the three treatment groups relative to NBTXR3+XRT+αPD1 in the secondary tumor. Two hundred and forty-six (246) such genes were identified and are here ranked alphabetically. **(C)** Genes identified as significantly upregulated in NBTXR3+XRT+PLT-treated mice compared to NBTXR3+XRT+αPD1-treated mice were manually grouped according to their established function. Functional groups were plotted according to the log_2_ fold change from baseline (NBTXR3+XRT+αPD1) of their constituent genes, represented in aggregate *via* violin plot.
