# Supplementary material for: Combining a nanoparticle-mediated immunoradiotherapy with dual blockade of LAG3 and TIGIT improves the treatment efficacy in anti-PD1 resistant lung cancer
Source: J Nanobiotechnology. 2022 Sep 19;20:417. doi: 10.1186/s12951-022-01621-4 (PMC9484155; doi:10.1186/s12951-022-01621-4)
Supplement: Supplementary file 2 — Additional file 2: Table S1. Log2 fold change and the function of the genes which were significantly up-regulated in the unirradiated tumors treated with NBTXR3+XRT+PLT vs. NBTXR3+XRT+αPD1. [file 12951_2022_1621_MOESM2_ESM.docx]

**Table S1.** Log2 fold change and the function of the genes which were significantly up-regulated in the unirradiated tumors treated with NBTXR3+XRT+PLT vs. NBTXR3+XRT+αPD1.

| **Gene** | **Log2 Fold Change** | **Full Name** | **Notable Aliases** | **Function** |
| --- | --- | --- | --- | --- |
| **Acute Phase Response** | | | | |
| *App* | 0.925 | Amyloid-beta precursor protein |  | A secreted antimicrobial peptide |
| *Cma1* | 1.15 | Chymase 1 | Mast cell protease 1 | Serine protease with pro-inflammatory peptidolytic activation predominantly secreted by mast cells |
| *Lbp* | 1.16 | Lipopolysaccharide binding protein |  | Soluble acute phase protein that binds to LPS and allows it to be recognized by CD14 and TLR4 |
| *Lcn2* | 1.84 | Lipocalin 2 | Neutrophil gelatinase-associated lipocalin (NGAL) | Neutrophil-secreted factor that sequesters iron-containing siderophores; also functions as a growth factor |
| *Psen1* | 1.04 | Presenilin-1 |  | Catalytic subunit of the gamma-secretase complex, which cleaves integral membrane proteins such as APP; also involved in calcium homeostasis |
| *Psen2* | 1.25 | Presenilin-2 |  | Putative catalytic subunit of the gamma-secretase complex, which cleaves integral membrane proteins such as APP; also involved in calcium homeostasis between the ER and the mitochondria |
| *Serping1* | 1.4 | Serpin family G member 1 | C1-inhibitor | Inflammation-induced acute phase protein that inhibits C1r and C1s proteases in the C1 complex |
| **Adhesion and cell-cell interactions** | | | | |
| *Cd47* | 0.972 | Cluster of differentiation 47 | Integrin-associated protein (IAP) | Partners with membrane integrins to serve as an inhibitor of phagocytosis |
| *Cd97* | 0.588 | Cluster of differentiation 97 | BL-Ac[F2] | GPCR that promotes granulocyte adhesion and migration; activates T cells *via* binding to CD55 |
| *Icam1* | 1.54 | Intracellular adhesion molecule 1 |  | Cell surface glycoprotein that serves as strong adhesive ligand for LFA-1; important for leukocyte mobility and costimulation |
| *Itga5* | 0.628 | Integrin alpha 5 |  | Pairs with ITGB1 to form a receptor for fibronectin and IL-1β |
| *Itgal* | 1.54 | Integrin alpha L |  | Pairs with ITGB2 to form lymphocyte function-associated antigen-1 (LFA-1), a common leukocyte adhesion molecule and costimulatory receptor |
| *Itgam* | 1.45 | Integrin alpha M | CD11b | Pairs with CD18 to forms Mac-1 *aka* complement receptor 3; mediates leukocyte activation, adhesion, chemotaxis, migration, phagocytosis, and cell-mediated cytotoxicity; serves as a macrophage marker |
| *Itgax* | 1.44 | Integrin alpha X | CD11c | Adhesion molecule; signature marker of antigen-presenting dendritic cells (DCs) |
| *Itgb2* | 1.63 | Integrin subunit beta 2 |  | Pairs with ITGAL to form a receptor for ICAM1, with ITGAM or ITGAX for iC3b and fibronectin |
| *Jam3* | 2.57 | Junctional adhesion molecule C |  | Immunoglobulin that mediates tight junctions between endothelial cells; mediates transepithelial migration of PMNs |
| *Sell* | 1.43 | L-selectin |  | Calcium-dependent lectin that mediates cell adhesion by binding to glycoproteins on neighboring cells |
| *Thy1* | 0.885 | Thy-1 T cell antigen |  | Cell surface glycoprotein involved in cell adhesion and communication in immune and nerve cells |
| **Apoptosis** | | | | |
| *Cyfip2* | 1.89 | Cytoplasmic FMR1-interacting protein 2 |  | Involved in T-cell adhesion and p53/TP53-dependent induction of apoptosis |
| *Fas* | 1.05 | Fragment apoptosis stimulating |  | Cell surface death receptor; interaction with FAS-ligand triggers an apoptotic signaling cascade; also activates NFκB, ERK1, and MAPK8 |
| *Ifi27* | 0.786 | Interferon alpha-inducible protein 27 |  | Sensitizes cells to apoptosis by embedding in the mitochrondria and releasing cytochrome C |
| *Ifitm2* | 0.819 | Interferon-induced transmembrane protein 2 |  | IFN-induced antiviral protein which inhibits the entry of viruses to the host cell cytoplasm; induces cell cycle arrest and mediates p53-independent apoptosis through caspase activation |
| *Trp53* | 0.507 | Transformation-related protein 53 |  | TF that induces cell-cycle arrest and apoptosis through stimulation of Fas expression |
| **Antigen Processing and Presentation** | | | | |
| *Cd1d1* | 1.57 | Antigen-presenting glycoprotein CD1d1 |  | Murine non-classical class I MHC; primarily presents lipid and glycolipid Ags |
| *Cd74* | 1.31 | Cluster of differentiation 74 | MHC class II gamma chain | Stabilizes peptide-free class II αβ heterodimers during MHC-Ag complex formation |
| *Cd83* | 1.9 | Cluster of differentiation 83 |  | APC surface marker; may be involved in the regulation of Ag presentation |
| *Clec4a2* | 1.25 | C-type lectin domain family 4 member a2 |  | PRR that, upon binding mannose or fucose, is endocytosed and processed in the Ag presentation pathway |
| *Ctsh* | 1.24 | Cathepsin H |  | Lysosomal protease; increased in macrophages in response to IFNγ |
| *Ctss* | 1.97 | Cathepsin S |  | Lysosomal protease that participates in processing of Ag by MHC class II |
| *H2-Aa* | 1.48 | H-2 class II histocompatibility antigen, A-B alpha chain |  | MHC class II molecule; presents Ags to CD4+ T cells |
| *H2-Ab1* | 1.44 | H-2 class II histocompatibility antigen, A beta chain |  | MHC class II molecule; presents Ags to CD4+ T cells |
| *H2-D1* | 0.943 | H-2 class I histocompatibility antigen, D-B alpha chain |  | MHC class I molecule; presents Ags to CD8+ T cells |
| *H2-DMa* | 1.49 | H-2 class I histocompatibility antigen, M alpha chain |  | MHC class I molecule; presents Ags to CD8+ T cells |
| *H2-K1* | 0.896 | H-2 class I histocompatibility antigen, K-B alpha chain |  | MHC class I molecule; presents Ags to CD8+ T cells |
| *H2-M3* | 0.943 | Histocompatibility 2, M region locus 3 |  | MHC class Ib molecule; presents Ags to CD8+ T cells, with a preference for N-formylated peptides |
| *H2-T23* | 0.927 | H-2 class I histocompatibility antigen, D-37 alpha chain |  | MHC class Ib molecule; presents Ags to CD8+ T cells |
| *Tap1* | 0.981 | Transporter antigen peptide 1 | Really interesting new gene 4 (RING4) | ATP-binding cassette transporter that pumps degraded cytosolic peptides from the cytosol to the ER for packaging into MHC class I molceules |
| **Autophagy** | | | | |
| *Atg12* | 0.773 | Autophagy related 12 |  | Pairs with ATG5 to promote the extension of the phagophoric membrane in autophagic vesicles |
| *Atg5* | 1.05 | Autophagy related 5 |  | Pairs with ATG12 to promote the extension of the phagophoric membrane in autophagic vesicles |
| *Irgm2* | 0.94 | Immunity-related GTPase family M member 2 | Interferon-inducible protein 1 (IFI1) | Function not fully known, but most likely regulates autophagy and pro-inflammatory cytokine production |
| *Ubc* | 1.17 | Polyubiquitin C |  | Serves various roles, including immate immunity, DNA repair, and stimulation of autophagy and the proteasomal response |
| **B Cell-associated Genes** | | | | |
| *Btk* | 1.41 | Bruton’s tyrosine kinase |  | Crucial kinase in BCR signal transmission and B cell activation |
| *Cd48* | 1.77 | Cluster of differentiation 48 | B-lymphocyte activation marker (BLAST-1); signaling lymphocytic activation molecule 2 (SLAMF2) | B cell-specific cellular differentiation antigen; when bound to CD2, promotes T cell activation, and the formation of lipid rafts and caveolae for macrophages |
| *Cxcl13* | 2.44 | C-X-C motif chemokine ligand 13 | BLC, BCA-1 | B cell chemokine induced by type I interferons; participates in germinal center formation |
| *Icosl* | 1.38 | Inducible T-cell costimulator ligand | CD275 | Ligand for T cell-specific co-receptor ICOS; also induces B cell proliferation and plasma cell differentiation |
| *Pou2f2* | 1.7 | POU domain class 2, transcription factor 2 |  | TF that regulates antibody and IL-6 expression in B cells |
| *Sh2d1a* | 1.45 | SH2 domain–containing protein 1A | SLAM-associated protein (SAP) | Adaptor protein involved in T, B, and NK cell signaling pathways; acts downstream of MHC class I and SLAMF7 |
| **Chemokines** | | | | |
| *Ccl4* | 1.9 | C-C motif chemokine ligand 4 | Macrophage inflammatory protein 1β (MIP1β) | Chemoattractant for NK cells and monocytes; binds to CCR5 receptors |
| *Ccl7* | 1.35 | C-C motif chemokine ligand 7 | Monocte chemotactic protein 3 (MCP3) | General chemokine that recruits leukocytes to infected tissues; mainly observed in monocyte mobilization |
| *Ccl8* | 1.7 | C-C motif chemokine ligand 8 | Monocyte chemoattractant protein 2 (MCP2) | General chemokine that recruits leukocytes to infected tissues |
| *Ccl12* | 1.32 | C-C motif chemokine ligand 12 | Monocyte chemotactic protein 5 (MCP5) | Chemoattractant specific for eosinophils, monocytes, and lymphocytes; found primarily in the lymph nodes and thymus, but can be strongly expressed by macrophages |
| *Ccl19* | 1.94 | C-C motif chemokine ligand 19 | Macrophage inflammatory protein-3 beta (MIP3β) | Chemokine that promotes normal lymphocyte recirculation and trafficking to the thymus and secondary lymphoid organs; binds to CCR7 |
| *Ccr2* | 1.51 | C-C motif chemokine receptor 2 | CD192 | Receptor for CCL2, a monocyte-specific chemokine |
| *Ccr7* | 1.83 | C-C chemokine receptor type 7 | CD197 | Chemokine receptor that activates B and T cells and promotes their homing to secondary lymphoid organs; also stimulates DC expression of MHC class I and II |
| *Ccrl2* | 1.46 | C-C chemokine receptor-like 2 |  | Stabilizes TLR4 surface expression on macrophages |
| *Cxcl10* | 1.12 | C-X-C motif chemokine ligand 10 | IFNγ-induced protein 10 (IP-10) | Macrophage, DC, T cell, and NK cell chemattractant secreted by several cell types in response to IFNγ; binds to CXCR3 |
| *Cxcl12* | 1.78 | C-X-C motif chemokine ligand 12 | Stromal cell-derived factor 1 (SDF1) | Ubiquitously expressed chemokine that acts a strong chemoattractant for lymphocytes |
| *Cxcl13* | 2.44 | C-X-C motif chemokine ligand 13 | BLC, BCA-1 | B cell chemokine induced by type I interferons; participates in germinal center formation |
| *Cxcl16* | 1.6 | C-X-C motif chemokine ligand 16 |  | Chemoattractant for T cells and NKT cells produced by DCs in response to IFNγ and TNFα |
| *Cxcr3* | 1.27 | C-X-C motif chemokine receptor 3 | CD183 | Induces integrin activation, cytoskeletal remodeling, and chemotaxis; expressed by T cells and NK cells; prominently expressed in effector and memory T cells |
| *Cxcr4* | 1.69 | Chemokine receptor CXCR4 | Fusin; CD184 | Alpha-chemokine receptor specific for SDF1 *aka* CXCL12 |
| **Cholesterol Metabolism** | | | | |
| *Abca1* | 1.73 | ATP-binding cassette transporter A1 |  | Membrane-associated cholesterol efflux pump |
| *Abcg1* | 1.75 | ATP-binding cassette transporter G1 |  | Membrane-associated cholesterol efflux pump |
| *Cd36* | 2.69 | Cluster of differentiation 36 | Fatty acid translocase (FAT) | Class B scavenger receptor that mediates fatty acid uptake |
| **Complement** | | | | |
| *C1qa* | 1.25 | Complement C1q subcomponent subunit A |  | A chain of the C1q complex, which acts as the Ag-Ab-binding subunit of the C1 complex |
| *C1qb* | 1.46 | Complement C1q subcomponent subunit B |  | B chain of the C1q complex, which acts as the Ag-Ab-binding subunit of the C1 complex |
| *C1ra* | 1.27 | Complement C1r-A subcomponent |  | Proteolytic subunit of the C1 complex that enzymatically cleaves C1s |
| *C1s1* | 1.49 | Complement component 1s |  | Serine protease that enzymatically cleaves C4 and C2 |
| *C2* | 1.91 | Complement component 2 |  | Serine protease that binds to C4b to form the C4bC2 complex |
| *C3* | 1.64 | Complement component 3 |  | Cleaved by C3 convertase to form C3a and C3b, an anaphalotoxin and an opsonizing agent, respectively |
| *C3ar1* | 1.07 | Complement component 3a receptor 1 |  | GPCR that binds to C3a, activating chemotaxis, granule enzyme release, superoxide anion production, and bacterial opsonization |
| *C4b* | 1.17 | Complement component 4B |  | Mediates interactions between antibody-bound antigens and other complement components |
| *C6* | 1.77 | Complement component 6 |  | Part of the membrane attack complex |
| *Cd55* | 2.74 | Cluster of differentiation 55 | Complement decay-accelerating factor | Cell surface glycoprotein that interacts with surface-bound C4b and inhibits its conversion of C2 to C2b |
| *Cfb* | 1.24 | Complement factor B |  | Alternate complement pathway component; when cleaved, produces a serine protease that binds to C3b to form C3 convertase |
| *Cfh* | 1.35 | Complement factor H |  | Soluble glycoprotein that regulates the alternate pathway by accelerating decay of C3 convertase |
| *Cfp* | 1.29 | Complement factor properdin |  | Alternate complement pathway component; when cleaved, produces a serine protease that binds to C3b to form C3 convertase |
| *Serping1* | 1.4 | Serpin family G member 1 | C1-inhibitor | Inflammation-induced acute phase protein that inhibits C1r and C1s proteases in the C1 complex |
| **Co-Stimulation** | | | | |
| *Cd27* | 0.824 | Cluster of differentiation 27 |  | Co-stimulatory receptor required for generation and long-term maintenance of T cell immunity |
| *Cd40* | 1.84 | Cluster of differentiation 40 |  | APC-expressed co-stimulatory protein that binds to CD40L on CD4+ T cells, causing activation of both |
| *Icosl* | 1.38 | Inducible T-cell costimulator ligand | CD275 | Ligand for T cell-specific co-receptor ICOS; also induces B cell proliferation and plasma cell differentiation |
| *Ptprc* | 1.5 | Protein tyrosine phosphatase receptor type C | CD45; leukocyte common antigen (LCA) | Delivers costimulation during T cell activation upon binding to its ligand DPP4; dephosphorylates Lyn and suppresses JAK kinases |
| **Cytokines** | | | | |
| *Csf1* | 1.03 | Macrophage colony-stimulating factor 1 |  | Cytokine that promote activation and survival of monocytes |
| *Csf1r* | 1.46 | Macrophage colony-stimulating factor 1 receptor |  | Receptor for CSF1; promotes release of inflammatory cytokines in response to IL-34 and CSF1 |
| *Ifnar1* | 0.997 | Interferon-alpha/beta receptor alpha chain |  | Component of the receptor for type I IFNs, binding of which activates the JAK-STAT pathway |
| *Ifng* | 1.37 | Interferon gamma |  | T and NK cell-secreted inflammatory cytokine that stimulates cytolysis, activates macrophages, and stimulates MHC class II expression |
| *Ifngr1* | 1.02 | Interferon gamma receptor 1 | CD54 | One of the two components of the IFNγ receptor; stimulates activation of the JAK/STAT signaling pathway |
| *Il1r1* | 0.735 | Interleukin 1 receptor type I | CD121a | Receptor for IL-1α and IL-1β; drives several cytokine-induced and inflammatory responses through activation of NFκB and MAPK; recruits TOLLIP, MyD88, IRAK1, and IRAK2 |
| *Il1rl1* | 0.549 | Interleukin 1 receptor-like 1 |  | Receptor for IL-33; recruits MyD88, IRAK1, IRAK4, and TRAF6; activates ERK1, ERK2, and MAPK14 |
| *Il2rg* | 1.76 | Interleukin 2 receptor subunit gamma | Common gamma chain; CD132 | Common subunit for the receptors for a variety of interleukins, including IL-2, -4, -7, and -21 |
| *Il4ra* | 0.818 | Interleukin 4 receptor subunit alpha | CD124 | Alpha chain for the IL-4 and IL-13 receptors; involved in TH2 differentiation and IgE production |
| *Il6* | 2.09 | Interleukin 6 |  | Pro-inflammatory cytokine that signals through the JAK and STAT pathways |
| *Il6st* | 0.961 | Interleukin 6 cytokine family signal transducer | Glycoprotein 130 (Gp130); CD130 | Transmembrane protein that acts a component in several cytokine receptors, including IL-6 |
| *Socs1* | 1.55 | Suppressor of cytokine signaling 1 |  | Inhibits JAK proteins; negative regulator of IL-6 |
| *Socs3* | 1.39 | Suppressor of cytokine signaling 3 |  | Inhibits IL6ST and JAK2; negative regulator of IL-6 |
| **Inflammation** | | | | |
| *Cma1* | 1.15 | Chymase 1 | Mast cell protease 1 | Serine protease with pro-inflammatory peptidolytic activation predominantly secreted by mast cells |
| *Ifng* | 1.37 | Interferon gamma |  | T and NK cell-secreted inflammatory cytokine that stimulates cytolysis, activates macrophages, and stimulates MHC class II expression |
| *Ifngr1* | 1.02 | Interferon gamma receptor 1 | CD54 | One of the two components of the IFNγ receptor; stimulates activation of the JAK/STAT signaling pathway |
| *Il6* | 2.09 | Interleukin 6 |  | Pro-inflammatory cytokine that signals through the JAK and STAT pathways |
| *Il6st* | 0.961 | Interleukin 6 cytokine family signal transducer | Glycoprotein 130 (Gp130); CD130 | Transmembrane protein that acts a component in several cytokine receptors, including IL-6 |
| *Irf1* | 1.19 | Interferon regulatory factor 1 |  | Transcriptional regulator that promotes inflammatory innate and adaptive immune responses |
| *Irf3* | 0.747 | Interferon regulatory factor 3 |  | Complexes with CREBBP to translocate to the nucleus and transcriptionally activate type I IFNs |
| *Irf4* | 2.16 | Interferon regulatory factor 4 |  | Transcriptional activator that complexes with BATF and binds ISREs within the promoters of multiple genes involved in inflammation |
| *Irf8* | 1.27 | Interferon regulatory factor 8 |  | TF that regulates of lineage commitment in myeloid cell maturation; promotes monocyte and plasmacytoid DC development |
| *F2rl1* | -0.941 | Coagulation factor II (thrombin) receptor-like 1 | Proteinase-activated receptor 2 (PAR2) | GPCR that, upon cleavage of its extracellular portion, becomes activated and initiates inflammatory signaling |
| *S100a8* | 2.11 | S100 calcium-binding protein A8 | Calgranulin A | One of the two components of calprotectin secreted by monocytes, granulocytes, and neutrophils during inflammation |
| *Tmem173* | 1.19 | Transmembrane protein 173 | Stimulator of interferon genes (STING) | Adaptor protein in type I IFN signaling; activates STAT6 and IRF3 through TBK1 to induce type I IFN production |
| *Vegfa* | 0.87 | Vascular endothelial growth factor A |  | Glysosylated mitogen that promotes vascular permeability, vasculogenesis, angiogenesis, and cell migration |
| **Inhibition** | | | | |
| *Btla* | 1.94 | B and T lymphocyte attenuator | CD272 | Inhibitory cell surface protein that inhibits T cell function by binding to B7H4 and TNFRSF14 |
| *Cd274* | 1.91 | Cluster of differentiation 274 | Programmed cell death receptor ligand 1 (PD-L1) | Ubiquitously expressed ligand for co-inhibitory receptor PD-1; upregulated by tumors as an immune evasion strategy |
| *Itch* | 0.652 | Itchy E3 ubiquitin protein ligase |  | Participates with TNFAIP3 in a ubiquitin-editing complex that marks components of inflammatory signaling pathways such as JUNB and CXCR4 for degradation |
| *Nfkb1* | 0.814 | Nuclear factor kappa B subunit 1 | p105/p50 | One of the NFκB family TFs; inhibits inflammation |
| *Nfkbia* | 1.32 | Nuclear factor kappa B inhibitor alpha |  | Inhibits activity of REL dimers by masking of their nuclear localization signals |
| *Socs1* | 1.55 | Suppressor of cytokine signaling 1 |  | Inhibits JAK proteins; negative regulator of IL-6 |
| *Socs3* | 1.39 | Suppressor of cytokine signaling 3 |  | Inhibits IL6ST and JAK2; negative regulator of IL-6 |
| *Tank* | 1.06 | TRAF family member-associated NFκB activator |  | Inhibitory protein that sequesters TRAFs in the cytoplasm, constitutively binds TBK1, and serves as a negative regulator of NFκB |
| *Tnfaip3* | 1.21 | Tumor necrosis factor |  | Inflammatory cytokine mainly produced by macrophages; binds to TNFRSF1A/TNFR1 and TNFRSF1B/TNFBR; capable of induce cell death of certain tumor cell lines |
| *Tnfrsf11a* | 1.2 | Tumor necrosis factor receptor superfamily member 11A | Receptor activator of NFκB (RANK) | Recruits TRAFs and activates NFκB and JNK activation upon binding to RANKL on the surface of T cells |
| **IRAKs & TRAFs** | | | | |
| *Irak1* | 0.827 | Interleukin-1 receptor-associated kinase 1 |  | Adaptor protein involved in TLR and IL-1 signaling; recruited to TLRs by MyD88 and phosphorylated by IRAK4; promotes the degradation of TIRAP |
| *Irak2* | 1.03 | Interleukin-1 receptor-associated kinase 2 |  | Adaptor protein involved in TLR and IL-1 signaling |
| *Irak3* | 1.19 | Interleukin-1 receptor-associated kinase 3 |  | Adaptor protein that negatively regulates TLR signaling; predominantly expressed in monocytes and macrophages |
| *Irak4* | 0.801 | Interleukin-1 receptor-associated kinase 4 |  | The primary IRAK family member in mammalian TLR and IL-1 signaling; joins with IRAK2 and MyD88 to form the myddosome complex to activate IRAK1 |
| *Traf3* | 0.482 | Tumor necrosis factor receptor-associated factor 3 |  | Adaptor protein that acts in the CD40 signaling cascade; induces NFκB and MAPK activation |
| *Traf6* | 0.956 | Tumor necrosis factor receptor-associated factor 6 |  | Adaptor protein that acts in the CD40 signaling cascade; promotes inflammation, IL-6, and TNFα |
| **JAK-STAT Pathway** | | | | |
| *Jak1* | 1.16 | Janus kinase 1 |  | Essential tyrosine kinase involved signal transduction in type I and II cytokines and IFNs |
| *Jak2* | 0.931 | Janus kinase 2 |  | Tyrosine kinase that participates in IFN and IL6ST signaling cascades |
| *Stat3* | 0.87 | Signal transducer and activator of transcription 3 |  | Transcriptional activator of genes involved in cell growth and apoptosis; activated by JAKs |
| *Stat4* | 0.996 | Signal transducer and activator of transcription 4 |  | Essential TF for TH1 CD4+ T cell development and IFNγ production; also promotes expression of MyD88 |
| *Stat6* | 0.859 | Signal transducer and activator of transcription 6 |  | Essential TF for TH2 CD4+ T cell and macrophage function and M2 macrophage polarization |
| **Macrophage-associated Genes** | | | | |
| *Camp* | 2.76 | Cathelicidin antimicrobial peptide |  | Polypeptide stored in the lysosomes of macrophages and PMNs that digests phagocytosed cells |
| *Ccl4* | 1.9 | C-C motif chemokine ligand 4 | Macrophage inflammatory protein 1β (MIP1β) | Chemoattractant for NK cells and monocytes; binds to CCR5 receptors |
| *Ccl12* | 1.32 | C-C motif chemokine ligand 12 | Monocyte chemotactic protein 5 (MCP5) | Chemoattractant specific for eosinophils, monocytes, and lymphocytes; found primarily in the lymph nodes and thymus, but can be strongly expressed by macrophages |
| *Ccl19* | 1.94 | C-C motif chemokine ligand 19 | Macrophage inflammatory protein-3 beta (MIP3β) | Chemokine that promotes normal lymphocyte recirculation and trafficking to the thymus and secondary lymphoid organs; binds to CCR7 |
| *Ccrl2* | 1.46 | C-C motif chemokine ligand 2 |  | Chemoattractant ligand for CCR2 and -4; attracts monocytes and basophils |
| *Cd14* | 1.49 | Cluster of differentiation 14 |  | PRR that recognizes LPS; mostly found on macrophages |
| *Cebpb* | 1.35 | CCAAT/enhancer-binding protein beta |  | Critical macrophage TF that promotes expresssion of several acute-phase and inflammatory cytokine genes, including *Il6* |
| *Csf1* | 1.03 | Macrophage colony-stimulating factor 1 |  | Cytokine that promote activation and survival of monocytes |
| *Csf1r* | 1.46 | Macrophage colony-stimulating factor 1 receptor |  | Receptor for CSF1; promotes release of inflammatory cytokines in response to IL-34 and CSF1 |
| *Ctsh* | 1.24 | Cathepsin H |  | Lysosomal protease; increased in macrophages in response to IFNγ |
| *Irak3* | 1.19 | Interleukin-1 receptor-associated kinase 3 |  | Adaptor protein that negatively regulates TLR signaling; predominantly expressed in monocytes and macrophages |
| *Itgam* | 1.45 | Integrin alpha M | CD11b | Pairs with CD18 to forms Mac-1 *aka* complement receptor 3; mediates leukocyte activation, adhesion, chemotaxis, migration, phagocytosis, and cell-mediated cytotoxicity; serves as a macrophage marker |
| *Marco* | 4.95 | Macrophage receptor with collagenous structure |  | A PRR that recognizes LDL |
| *Slamf7* | 2.19 | Signaling lymphocytic activation molecule family member 7 |  | A super-activator of macrophages and a strong promoter of phagocytosis; binds to CD74 |
| *Slc11a1* | 2.04 | Natural resistance-associated macrophage protein 1 |  | Macrophage-specific metal ion transporter; uptakes divalent metal cations to neutralize ROSs |
| *Stat6* | 0.859 | Signal transducer and activator of transcription 6 |  | Essential TF for TH2 CD4+ T cell and macrophage function and M2 macrophage polarization |
| **MAP Kinase Signaling** | | | | |
| *Dusp6* | 0.708 | Dual specificity phosphatase 6 |  | Inhibitively phosphorylates ERK1 and 2 |
| *Map2k1* | 0.534 | Dual specificity mitogen-activated protein kinase kinase 1 | MAPK/ERK kinase 1 (MEK1) | Essential component of the MAP kinase signal transduction pathway; participates in numerous biological functions, including cell growth, adhesion, survival, differentiation, transcription, metabolism, and cytoskeletal remodeling |
| *Map3k7* | 0.896 | Mitogen-activated protein kinase kinase kinase 7 | TGFβ-activated kinase (TAK1) | Signal transducer downstream of TGFβ and BMP; controls a variety of cell functions, including transcription regulation and apoptosis |
| *Map4k2* | 1.19 | Mitogen-activated protein kinase kinase kinase kinase 2 |  | Essential component of the MAP kinase signal transduction pathway downstream of TRAF6; upstream activator of the SAP/JNK signaling pathway; |
| *Mapk14* | 0.97 | Mitogen-activated protein kinase 14 |  | One of the four p38 MAPKs; key kinase in the cascades of cellular responses evoked by extracellular stimuli such as proinflammatory cytokines |
| *Mapkapk2* | 1.21 | MAP kinase-activated protein kinase 2 |  | Serine/threonine-protein kinase involved in cytokine production, endocytosis, reorganization of the cytoskeleton, cell migration, cell cycle control, chromatin remodeling, DNA damage response, and transcriptional regulation |
| **NFκB Signaling** | | | | |
| *Bcl10* | 0.966 | B cell lymphoma/leukemia 10 |  | Activates NFκB *via* ubiquitination of IKKγ |
| *Chuk* | 0.604 | Conserved helix-loop-helix ubiquitous kinase | Inhibitor of NFκB kinase subunit alpha (IKKα) | Part of the IKK complex that inhibits IκBα and permits NFκB nuclear localization |
| *Ikbkb* | 0.551 | Inhibitor of nuclear factor kappa B kinase subunit beta |  | Part of the IKK complex that inhibits IκBα and permits NFκB nuclear localization |
| *Nfkb1* | 0.814 | Nuclear factor kappa B subunit 1 | p105/p50 | One of the NFκB family TFs; inhibits inflammation |
| *Nfkbia* | 1.32 | Nuclear factor kappa B inhibitor alpha |  | Inhibits activity of REL dimers by masking of their nuclear localization signals |
| *Rel* | 1.48 | Avian reticuloendotheliosis viral oncogene homolog | c-Rel | One of the NFκB family TFs; important for B cell and Treg development |
| *Rela* | 0.708 | Avian reticuloendotheliosis viral oncogene homolog A | p65 | One of the NFκB family TFs; major driver of inflammation |
| *Relb* | 1.19 | Avian reticuloendotheliosis viral oncogene homolog B |  | One of the NFκB family TFs; controls lymphoid development, DC biology, and noncanonical NFκB signaling |
| **Phagocytosis** | | | | |
| *Camp* | 2.76 | Cathelicidin antimicrobial peptide |  | Polypeptide stored in the lysosomes of macrophages and PMNs that digests phagocytosed cells |
| *Cd47* | 0.972 | Cluster of differentiation 47 | Integrin-associated protein (IAP) | Partners with membrane integrins to serve as an inhibitor of phagocytosis |
| *Mertk* | 1.95 | Monocytes and tissues of epithelial and reproductive orgin tyrosine kinase |  | Receptor tyrosine kinase that governs cell survival, platelet aggregation, cytoskeletal reorganization, migration, differentiation, and efferocytosis |
| *Slamf7* | 2.19 | Signaling lymphocytic activation molecule family member 7 |  | A super-activator of macrophages and a strong promoter of phagocytosis; binds to CD74 |
| **ROS Generation** | | | | |
| *Cybb* | 2.26 | Cytochrome b-245 heavy chain | Nox2 | Part of the NADPH oxidase process; generates superoxides |
| *Dpp4* | 1.25 | Dipeptidyl-peptidase 4 | CD26 | Serine exopeptidase that cleaves various substrates, thereby inactivating them |
| *Nos2* | 3.29 | Inducible nitric oxide synthase (iNOS) |  | Produces reactive oxygen species and contributes to inflammatory cytokine production |
| *Txnip* | 1.27 | Thioredoxin interacting protein |  | Thiol-oxidoreductase; protects cells from oxidative stress by inhibiting thioredoxin |
| **T Cell Receptor Signaling** | | | | |
| *Cd3d* | 1.28 | T cell surface glycoprotein CD3 delta chain |  | Component of the TCR-CD3 complex; upon phosphorylation by Lck, serves as a docking station for downstream TCR signaling adaptors |
| *Cd4* | 1.43 | Cluster of differentiation 4 |  | Signature helper T cell marker; binds to MHC class II and provides necessary costimulation for T cell activation |
| *Cd247* | 1.22 | T cell surface glycoprotein CD3 zeta chain |  | Central intracellular signaling chain of the TCR, to which downstream signaling adaptors dock |
| *Lcp1* | 1.31 | Lymphocyte cytosolic protein 1 | Plastin-2 | Actin-binding protein that promotes T cell activation in response to costimulation through TCR/CD3 and CD2 or CD28; assists with IL2RA transport to the cell surface |
| *Lck* | 1.27 | Lymphocyte cell kinase |  | Src family tyrosing kinase that acts as one of the main signaling intermediaries downstream of the TCR; constitutively associated with the cytoplasmic portion of CD4 |
| *Nfatc1* | 0.811 | Nuclear factor of activated T cells, cytoplasmic 1 |  | Inducible nuclear component of the NFAT TF complex; mediates induction of IL-2 and IL-4 in T cells |
| *Nfatc2* | 1.25 | Nuclear factor of activated T cells, cytoplasmic 2 |  | Cytosolic component of the NFAT TF complex; mediates induction of IL-2, IL-3, IL-4, TNFα, and GM-CSF |
| *Nfatc4* | 0.905 | Nuclear factor of activated T cells, cytoplasmic 4 |  | TF that mediates induction of IL-2 and IL-4 in T cells; possibly involved in APP processing |
| *Pik3cd* | 1.41 | Phosphatidylinositol-4,5-bisphosphate 3-kinase catalytic subunit delta isoform |  | A subunit of PI3K; acts downstream of TLR4, TCR, BCR, and CD40; contributes to T helper cell expansion, mast cell development, and neutrophil chemotaxis, extravasation, and respiratory burst |
| *Pik3cg* | 1.57 | Phosphatidylinositol-4,5-bisphosphate 3-kinase catalytic subunit gamma isoform |  | A subunit of PI3K; modulates leukocyte chemokine-driven homing |
| *Sh2d1a* | 1.45 | SH2 domain–containing protein 1A | SLAM-associated protein (SAP) | Adaptor protein involved in T, B, and NK cell signaling pathways; acts downstream of MHC class I and SLAMF7 |
| *Spn* | 1.07 | Sialophorin | Leukosialin; CD43 | Cell surface sialoglycoprotein expressed by T cells, B cells, monocytes, and granulocytes; promotes lymph node localization in T cells; shunts T cells away from the TH2 phenotype and towards TH1; promotes the expression of IFNγ in CD4+ T cells |
| *Syk* | 1.75 | Spleen-associated tyrosine kinase |  | Critical kinase that transmits signals from the TCR and BCR |
| *Tcf7* | 1.49 | Transcription factor 7 |  | HMG box TF predominantly expressed by T cells that drives their development, although also involved in NK cell development; activates transcription through a Wnt/β-catenin signaling pathway |
| **Toll-like Receptors & Other PRRs** | | | | |
| *Cd14* | 1.49 | Cluster of differentiation 14 |  | PRR that recognizes LPS; mostly found on macrophages |
| *Cd180* | 1.92 | Cluster of differentiation 180 |  | Heterodimeric binding partner of MD-1 that participates in LPS binding in APCs |
| *Ddx58* | 0.84 | DExD/H-box helicase 58 | Retinoic acid-inducible gene I (RIG-I) | Cytoplasmic PRR that recognizes dsRNA; can promote T cell-independent B cell activation; uses MAVS as an adaptor |
| *Ly86* | 1.68 | Lymphocyte antigen 86 | Myeloid differentiation factor 1 (MD-1) | Heterodimeric binding partner of CD180 that participates in LPS binding in APCs |
| *Ly96* | 1.19 | Lymphocyte antigen 96 | Myeloid differentiation factor 2 (MD-2) | Heterodimeric binding partner of TLR4 that participates in LPS binding |
| *Marco* | 4.95 | Macrophage receptor with collagenous structure |  | A PRR that recognizes LDL |
| *Myd88* | 0.962 | Myeloid differentiation primary response 88 |  | Key adaptor in the TLR signaling pathways; interacts with all TLRs except TLR3; activates NFκB and IRFs |
| *Ticam2* | 1.64 | TIR domain-containing adaptor molecule 2 |  | Sorting adapter in various innate immune signaling cascades; bridges TLR2 and MyD88 |
| *Tlr2* | 1.51 | Toll-like receptor 2 | CD282 | Surface PRR that binds to various lipid-containing PAMPs |
| *Tlr3* | 0.715 | Toll-like receptor 3 | CD283 | Endosomic PRR that recognizes dsRNA |
| *Tlr7* | 1.83 | Toll-like receptor 7 | CD287 | Endosomic PRR that recognizes ssRNA |
| *Tlr8* | 1.58 | Toll-like receptor 8 | CD288 | Endosomic PRR that recognizes ssRNA |
| *Tollip* | 0.703 | Toll interacting protein |  | Inhibitory adaptor protein that acts upon TLR2 |
| **Transcription Factors and Chromatin Remodellers** | | | | |
| *Atf1* | 0.84 | Cyclic AMP-dependent transcription factor |  | Basic leucine zipper TF regulating the expression of genes related to growth and survival |
| *Bcl6* | 1.32 | B cell lymphoma 6 transcriptional repressor |  | Zing finger TF required for GC and memory formation in both B and T cells |
| *Cebpb* | 1.35 | CCAAT/enhancer-binding protein beta |  | Critical macrophage TF that promotes expresssion of several acute-phase and inflammatory cytokine genes, including *Il6* |
| *Egr1* | 1.46 | Early growth response 1 | Zinc finger protein 268 (ZNF286) | Transcriptional repressor of genes involved in differentiations and mitogenesis; activates expression of p53 |
| *Ep300* | 0.73 | Adenovirus early region 1A-associated protein p300 |  | Histone acetyltransferase; participates in chromatin remodeling to facilitate gene accessibility |
| *Fos* | 1.08 | Finkel-Biskel-Jenkins osteosarcoma viral oncogene homolog |  | Basic leucine zipper TF that dimerizes with JUN proteins to form the AP-1 TF complex |
| *Hmgb1* | 0.481 | High-mobility group box 1 |  | Remodels chromatin to make DNA more available for transcription |
| *Ikzf2* | 0.833 | IKAROS family zinc finger protein 2 |  | Heamtopoietic cell-specific TF involved in early hematopoietic development |
| *Nfatc1* | 0.811 | Nuclear factor of activated T cells, cytoplasmic 1 |  | Inducible nuclear component of the NFAT TF complex; mediates induction of IL-2 and IL-4 in T cells |
| *Nfatc2* | 1.25 | Nuclear factor of activated T cells, cytoplasmic 2 |  | Cytosolic component of the NFAT TF complex; mediates induction of IL-2, IL-3, IL-4, TNFα, and GM-CSF |
| *Nfatc4* | 0.905 | Nuclear factor of activated T cells, cytoplasmic 4 |  | TF that mediates induction of IL-2 and IL-4 in T cells; possibly involved in APP processing |
| *Pou2f2* | 1.7 | POU domain class 2, transcription factor 2 |  | TF that regulates antibody and IL-6 expression in B cells |
| *Tcf7* | 1.49 | Transcription factor 7 |  | HMG box TF predominantly expressed by T cells that drives their development, although also involved in NK cell development; activates transcription through a Wnt/β-catenin signaling pathway |
| *Yy1* | 0.519 | Yin yang 1 |  | Ubiquitous factor that serves as a transcriptional "switch", either promoting or repressing the transcription of numerous genes through the selective recruitment of either histone deacetylases or acetyltransferases; plays a fundamental role in diverse processes, such as differentiation, replication, and cellular proliferation |
| **Tyrosine Kinases** | | | | |
| *Btk* | 1.41 | Bruton’s tyrosine kinase |  | Crucial kinase in BCR signal transmission and B cell activation |
| *Hck* | 1.55 | Hematopoietic cell kinase |  | Src family tyrosine kinase that mediates secretory lysosome mobilization, degranlation, and activation of NADPH oxidase |
| *Lck* | 1.27 | Lymphocyte cell kinase |  | Src family tyrosing kinase that acts as one of the main signaling intermediaries downstream of the TCR; constitutively associated with the cytoplasmic portion of CD4 |
| *Lyn* | 1.18 | Tyrosine-protein kinase Lyn |  | Src family tyrosine kinase that potentiates signaling from the B cell receptor and CD40 |
| *Syk* | 1.75 | Spleen-associated tyrosine kinase |  | Critical kinase that transmits signals from the TCR and BCR |
| *Tbk1* | 0.945 | TANK-binding kinase 1 |  | Coordinates the activation of IRF3 and NFκB and induction of type I IFNs |
| **Ubiquitinases** | | | | |
| *Tnfaip3* | 1.21 | Tumor necrosis factor, alpha-induced protein 3 |  | Ubiquitin-editing enzyme that complexes with ITCH to degrade inflammatory signaling components in the TNF, IL1, and TLR pathways; targets TRAF2, TRAF6, and IKK |
| *Tnfrsf11a* | 1.2 | Tumor necrosis factor receptor superfamily member 11A | Receptor activator of NFκB (RANK) | Recruits TRAFs and activates NFκB and JNK activation upon binding to RANKL on the surface of T cells |
| **Other** | | | | |
| *Ada* | 0.921 | Adenosine deaminase |  | Key enzyme in purine metabolism; primarily involved in the development and maintenance of the immune system in humans |
| *Ccnd3* | 1.01 | Cyclin D3 |  | Regulatory component of the cyclin D3-CDK4 complex that inhibitively phosphorylates members of the retinoblastoma protein family; regulates the cell-cycle during G(1)/S transition |
| **Legend:  Ab** - antibody; **Ag** - antigen; **APC** - antigen presenting cell; **BCR** - B cell receptor; **BMP** - bone morphogenic protein; **DC** - dendritic cell; **LDL** - low density lipoprotein; **GPCR** - G protein-coupled receptor; **HMG** - High mobility group; **IFN** - interferon; **PAMPs** - pathogen-associated molecular patterns; **PMN** - polymorphonuclear leukocytes, *aka* granulocytes; **PRR** - pattern recognition receptor; **ROS** - reactive oxygen species; **TCR** - T cell receptor; **TF** - transcription factor | | | | |
